# Adverse effect signature extraction and prediction for drugs treating COVID-19

**DOI:** 10.3389/fgene.2022.1019940

**Published:** 2022-11-04

**Authors:** Han Wang, Xin Wang, Teng Li, Daoyuan Lai, Yan Dora Zhang

**Affiliations:** ^1^ Department of Statistics and Actuarial Science, The University of Hong Kong, Hong Kong SAR, China; ^2^ Department of Medical Oncology, National Cancer Center/National Clinical Research Center for Cancer/Cancer Hospital, Chinese Academy of Medical Sciences and Peking Union Medical College, Beijing, China; ^3^ Centre for PanorOmic Sciences, The University of Hong Kong, Hong Kong SAR, China

**Keywords:** COVID-19 drugs, adverse effect signature, non-negative matrix factorization, network integration, precise drug recommendation

## Abstract

Given the considerable cost of drug discovery, drug repurposing is becoming attractive as it can effectively shorten the development timeline and reduce the development cost. However, most existing drug-repurposing methods omitted the heterogeneous health conditions of different COVID-19 patients. In this study, we evaluated the adverse effect (AE) profiles of 106 COVID-19 drugs. We extracted four AE signatures to characterize the AE distribution of 106 COVID-19 drugs by non-negative matrix factorization (NMF). By integrating the information from four distinct databases (AE, bioassay, chemical structure, and gene expression information), we predicted the AE profiles of 91 drugs with inadequate AE feedback. For each of the drug clusters, discriminant genes accounting for mechanisms of different AE signatures were identified by sparse linear discriminant analysis. Our findings can be divided into three parts. First, drugs abundant with AE-signature 1 (for example, remdesivir) should be taken with caution for patients with poor liver, renal, or cardiac functions, where the functional genes accumulate in the RHO GTPases Activate NADPH Oxidases pathway. Second, drugs featuring AE-signature 2 (for example, hydroxychloroquine) are unsuitable for patients with vascular disorders, with relevant genes enriched in signal transduction pathways. Third, drugs characterized by AE signatures 3 and 4 have relatively mild AEs. Our study showed that NMF and network-based frameworks contribute to more precise drug recommendations.

## Introduction

The coronavirus disease 2019 (COVID-19) has swept the world for over 2 years. Although COVID-19 vaccines have made an indelible contribution to triumphing over the epidemic, it is not the silver bullet to end the pandemic. An increasing number of infected cases were reported even though they were fully vaccinated (i.e., the COVID-19 vaccine breakthrough infections) (CDC Covid-19 Vaccine Breakthrough Case Investigations Team, 2021). There is an urgent need to develop therapeutic drugs to fight against this pandemic.

Because traditional drug discovery methods are time consuming and expensive, the drug repurposing method is becoming an attractive option in the current urgent circumstance. Recently, many computational approaches have been developed to narrow down the search space of drugs to accelerate the process of drug repurposing against SARS-COV-2 (Gordon et al., 2020; Jang et al., 2021; Adhami et al., 2021; Guo et al., 2021; Gysi et al., 2021). Among them, the network-based approach accounts for a large proportion. Rintala et al. (2022) reviewed some representative drug repurposing results for COVID-19, which applied network proximity algorithms. Most of the existing computational methods have two main shortcomings. First, only one particular type of data was used in the drug repurposing procedure, which may limit the power to identify therapeutic candidates. Integrating multiple heterogeneous data sources would be more powerful. The other shortcoming is the so-called winner-takes-all pattern. Specifically, the selected repurposed drug may be recommended to all patients without taking the heterogeneous health conditions into account. In the era of precision medicine, it is normal to recommend specific drugs for patients with distinct underlying conditions, even though they are diagnosed with the same disease. To address the limitations of the winner-takes-all pattern in the existing drug repurposing scheme, predicting the drug’s heterogeneous safety levels with respect to the different health conditions of patients is essential.

From the perspective of drug safety, drugs repurposed for treating COVID-19 can be classified into two categories. The first category contains drugs that are still in or about to be in the stage of clinical trials. Because these drugs have not been used by COVID-19 patients on a large scale, their adverse effect (AE) feedback may be inadequate and the evaluation of drug safety remains unclear. In this case, it is more desirable to conduct computational methods to predict side effects for drugs in the clinical trial stage to save research time and cost. The prediction of drug side effects is usually based on drug–drug similarity through integrating multi-source data, including chemical structures, protein targets, and therapeutic indication (Wang et al., 2014; Zhang et al., 2016; Timilsina et al., 2019).

The second category involves drugs that have been approved by the World Health Organization (WHO) or drugs with relatively abundant post-market surveillance-reported AEs offered by COVID-19 patients. Jing et al. (2021) mined the AE information of several COVID-19 drugs using the FDA adverse events reporting system (FAERS) database, presenting the landscape of overreported AEs in each organ/system for each drug. Wu et al. (2020) explored the associations between COVID-19 drugs and 30 human tissues based on network proximity. Although it is more systematic to classify AEs into tissue level or system level, the biological process causing AEs might be cross organs or cross systems. Thus, it is more powerful to integrate different AEs and different drugs together to dig out a higher-level landscape of AEs, which can give us a comprehensive picture of drug safety and offer benefits for drug recommendation in precision medicine.

In this drug safety study, we conducted the AE profile for two different kinds of drugs: drugs with abundant AE information and drugs without abundant AE information. First, based on the relatively abundant AE information provided by FAERS, we proposed a non-negative matrix factorization (NMF) (Lee and Seung, 2000) framework for drugs that have already been used to treat COVID-19. Apart from extracting four AE signatures to depict each drug’s AE distribution, NMF also provided abundance fractions on the four AE signatures for each drug, which we refer to a characteristic combination of AEs as an AE-signature. The higher-level landscape of AEs represented by AE signatures can help us partition the whole COVID-19 patient cohort into four subpopulations, with each subpopulation more vulnerable to its corresponding AE signature. Therefore, we can conduct the precise drug recommendation by incorporating the AE signature’s abundance fraction for each drug.

Second, because COVID-19 drugs in the clinical trial stage lack AE feedback, we predicted their AE profiles to achieve more accurate personalized drug recommendations. Specifically, we

1) Constructed drug–drug similarity networks using data coming from multiple sources
2) Built a network imputation framework to tackle information imbalance among networks
3) Combined the integrated similarity network with drugs possessing a relatively affluent drug safety profile

We presented literature support and molecular-level explanations to illustrate the rationality of the AE prediction results.

## Methods

### Workflow

**FIGURE 1:**
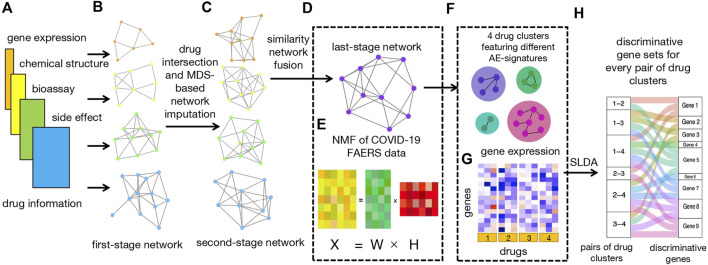
Workflow. **(A)** Four types of input data, including bioassay, chemical structure, LINCS, and FAERS data. **(B)** Construction of four first-stage networks based on four types of drug data. **(C)** Construction of four second-stage networks based on drug intersection and MDS-based network imputation. **(D)** Construction of last-stage network from similarity network fusion (SNF). **(E)** Non-negative matrix factorization (NMF) of count matrix from the CEUAFD; X , drug-AE count matrix; W , AE-signature matrix; and H , drug abundance fraction matrix. **(F)** Clustering of all COVID-19 drugs by combining results from **(D)** and **(E)**, with each cluster featuring one AE signature. **(G)** Gene expression information extracted from LINCS data with the A549 cell line for four clusters of drugs. **(H)** Discriminant gene sets obtained by sparse linear discriminant analysis (SLDA) with respect to each pair of drug clusters.

The workflow of our study is illustrated in Figure 1.

First of all, we collected drugs that are in the current COVID-19 clinical trial stage (Pundi et al., 2020; Bugin and Woodcock, 2021) together with drugs from the COVID-19 Emergency Use Authorization FAERS database (CEUAFD), mined their information from four different datasets (encompassing AE, bioassay, chemical structure, and gene expression information), and constructed four first-stage drug–drug similarity networks accordingly (see Supplementary Material). For simplicity, we refer to COVID-19 drugs that emerged in the CEUAFD as CEUAFD drugs hereafter. Second, for drugs in the clinical trial stage, we only reserved drugs whose information was available among all four databases. For some CEUAFD drugs abundant in certain AE signatures, their information across all four databases may be incomplete. To leverage their informative AE feedback and tackle the information imbalance problem, we built an imputation method based on multi-dimensional scaling (MDS) (Cox and Cox, 2008) to patch up the information imbalance among data sources, forming four-dimension-consistent imputed second-stage networks. Third, we applied the similarity network fusion (SNF) method (Wang et al., 2014) to the four second-stage networks to obtain a final-stage integrated drug–drug similarity network (see Supplementary Material). At the same time, we conducted NMF to the CEUAFD drugs to extract four higher-level AE signatures and drugs’ abundance fractions on the deciphered AE signatures. Meanwhile, the dimension reduction of AEs and clustering of CEUAFD drugs can be achieved. Finally, based on the integrated similarity network and CEUAFD drugs’ clustering outcome from the NMF procedure, we obtained four drug clusters for all analyzed COVID-19 drugs, with each cluster featuring one specific AE signature. Precise recommendations for drugs in different clinical stages can be implemented subsequently (see Supplementary Material). To demonstrate the reliability of our predicted results, we performed extensive literature explorations and measured drug–tissue distances by taking a network proximity approach on the Genotype-Tissue Expression (GTEx) database (Lonsdale et al., 2013). For every pair of drug clusters among the four clusters, we conducted sparse linear discriminant analysis (SLDA) (Mai et al., 2012) to pick up genes with discriminant expression patterns. If there is a correspondence between discriminant genes and AE signatures for each pair of drug clusters, the rationality of our drug clustering results can be proved. Pathway enrichment analysis (see Supplementary Material) and gene function analysis revealed that the discriminant genes do have the ability to account for different AE signatures in each pair of drug clusters.

### Network imputation

We found that drugs abundant with AE feedback may have insufficient information from other data sources (for example, multi-compound drugs casirivimab and imdevimab). To make full use of the AE information reported by COVID-19 patients and construct a more informative integrated drug–drug similarity network, we imputed similarity scores for the other three databases based on the FAERS data.

We took the bioassay database as an example to demonstrate the imputation process. Suppose the first-stage drug–drug similarity matrices constructed by FAERS and bioassay database are denoted by $S_{f}\in R^{n_{f}\times n_{f}}$ and $S_{a}\in R^{n_{a}\times n_{a}}$ (see Supplementary Material). To make drug information reflected by different databases transmissible, we adopted the MDS (Cox and Cox, 2008) method to translate “drug–drug similarity” into “lower-dimensional coordinate” for all drugs. Therefore, a predictive model linking the “lower-dimensional coordinate” of FAERS and the bioassay database can be built using dataset-shared drugs. For drugs that lack information in bioassay form, the information provided by the FAERS database can be transmitted by this predictive model to fulfill the imputation.

To let the “lower-dimensional coordinate” of drugs preserve the similarity given by $S_{f}$ or $S_{a}$ to the largest extent, we applied an eigenvalue decomposition (EVD) method to the double-centering matrix $C_{f}=-\frac{1}{2}H_{f}S_{f}H_{f}$ and $C_{a}=-\frac{1}{2}H_{a}S_{a}H_{a}$ , where $H_{f}$ and $H_{a}$ are the centering matrix, i.e., $H_{f}=I_{n_{f}}-J_{n_{f}}/n_{f}$ , and $H_{a}=I_{n_{a}}-J_{n_{a}}/n_{a}$ with $I_{n_{f}}\in R^{n_{f}\times n_{f}}$ and $I_{a}\in R^{n_{a}\times n_{a}}$ denoting the identity matrix and $J_{n_{f}}\in R^{n_{f}\times n_{f}}$ and $J_{a}\in R^{n_{a}\times n_{a}}$ denoting the matrix of all ones. Concretely, we suppose

$$C_{f}=P\Lambda_{f}P^{T}≜\tilde{P}{\tilde{P}}^{T},$$ (1)

$$C_{a}=Q\Lambda_{a}Q^{T}≜\tilde{Q}{\tilde{Q}}^{T},$$ (2)

where P and Q are orthogonal matrices with $P=\left(\xi_{1},...,\xi_{n_{f}}\right)$ and $Q=\left(\eta_{1},...,\eta_{n_{a}}\right).$ Here, we denote $\xi_{k}={\left(\xi_{k,1},...,\xi_{k,n_{f}}\right)}^{T}$ for $k=1,...,n_{f}$ ; $\eta_{l}={\left(\eta_{l,1},...,\eta_{l,n_{a}}\right)}^{T}$ for $l=1,...,n_{a}$ . $\Lambda_{f}$ and $\Lambda_{a}$ are diagonal matrices with $\Lambda_{f}=diag\left(\lambda_{f,1},...,\lambda_{f,n_{f}}\right)$ , $\Lambda_{a}=diag\left(\lambda_{a,1},...,\lambda_{a,n_{a}}\right).$ $\tilde{P}=P\Lambda_{f}^{\frac{1}{2}}=\left(\sqrt{\lambda_{f,1}}\xi_{1},...,\sqrt{\lambda_{f,n_{f}}}\xi_{n_{f}}\right)$ and $\tilde{Q}=Q\Lambda_{a}^{\frac{1}{2}}=\left(\sqrt{\lambda_{a,1}}\eta_{1},...,\sqrt{\lambda_{a,n_{a}}}\eta_{n_{a}}\right)$ . For convenience, we suppose $\lambda_{f,1}\geq ...\geq \lambda_{f,n_{f}}\geq 0$ , $\lambda_{a,1}\geq ...\geq \lambda_{a,n_{a}}\geq 0$ .

If the $n_{f}$ and $n_{a}$ drugs in FAERS and bioassay databases are embedded in the $k_{f}$ - and $k_{a}$ -dimensional Euclidean space separately, the coordinates of drugs in the two datasets can be represented by ${\tilde{P}}_{k_{f}}=\left(\sqrt{\lambda_{f,1}}\xi_{1},...,\sqrt{\lambda_{f,k_{f}}}\xi_{k_{f}}\right)$ and ${\tilde{Q}}_{k_{a}}=\left(\sqrt{\lambda_{a,1}}\eta_{1},...,\sqrt{\lambda_{a,k_{a}}}\eta_{k_{a}}\right)$ , with each row denoting one drug’s coordinate. More specifically, the coordinate of the i -th drug in the FAERS database is $\left(\sqrt{\lambda_{f,1}}\xi_{1,i},...,\sqrt{\lambda_{f,k_{f}}}\xi_{k_{f},i}\right)$ , and the coordinate of the j -th drug in the bioassay database is $\left(\sqrt{\lambda_{a,1}}\eta_{1,j},...,\sqrt{\lambda_{a,k_{a}}}\eta_{k_{a},j}\right)$ .

We introduce set U to contain drugs shared between FAERS and bioassay datasets. We denote the coordinates in these two datasets as $P_{U}\in R^{\left|U\right|\times k_{f}}$ and $Q_{U}\in R^{\left|U\right|\times k_{a}}$ separately and assume a linear relationship between $P_{U}$ and $Q_{U}$ , i.e.,

$$Q_{U}=P_{U}B+E,$$ (3)

where B is the coefficient matrix and $E\in R^{\left|U\right|\times k_{f}}$ is the noise matrix with every element $e_{i,j}∼N\left(0,\sigma^{2}\right)$ . If we denote the least square estimator of B as $\hat{B}$ , for a particular drug only equipped with FAERS coordinates ζ while lacking bioassay coordinates, we can predict its bioassay coordinate by $\zeta^{T}\hat{B}$ . Once the bioassay coordinates are predicted, the similarity score in the bioassay database can be computed by the Euclidean distance between the bioassay coordinates of drugs.

### Non-negative matrix factorization of COVID-19 emergency use authorization FAERS database drugs

Suppose there are n unique drugs and p unique AEs reported in the CEUAFD after the pre-processing process (see Supplementary Material), and $X\in R^{p\times n}$ is a matrix with element $X_{ij}$ representing the total number of patients reporting the i -th AE after taking the j -th drug ( i=1 ,..., p and j=1 ,..., n ). We applied NMF to X to decipher the underlying AE signatures among CEUAFD drugs. Meanwhile, for each drug, we assigned an abundance score to each AE signature. Concretely, X is approximated by the product of two low-rank matrices:

X≈WH, (4)

where $W\in R^{p\times k}$ is the AE-signature matrix with each column corresponding to a specific AE signature, $H\in R^{k\times n}$ is an abundance fraction matrix with the j -th column representing the relative abundance on each AE signature for the j -th drug ( j=1 ,..., n ), and k is the number of AE signatures. After data pre-processing, we have p = 134, n = 15.

We adopted the optimization algorithm given by Lee and Seung (2000) to get the factorized matrix W and H . In practice, we proceeded NMF with 50 different initializations of W and H to avoid sticking in the local stationary point.

### Clustering of CEUAFD drugs

Similar to the work of Brunet et al. (2004), H returned by the NMF procedure can be used for drug clustering. If $v^{*}=\underset{v=1,...,k}{\text{argmax}}h_{vj}$ , we can classify drug j into cluster $v^{*}$ .

### Tissue-specific genes in the GTEx database

For a specific tissue t , we denote the mean expression level for gene g as ${\bar{E}}_{tg}$ , and the mean expression level for gene g across all tissues in the GTEx database as ${\bar{E}}_{g}$ . We further assume that among all tissues, the standard deviation of the expression level for gene g is $S_{g}$ , and denote the tissue-specific score for gene g in tissue t as

$$Z_{tg}=\frac{{\bar{E}}_{tg}-{\bar{E}}_{g}}{S_{g}}.$$ (5)

For each tissue t , we pick up 200 tissue-specific genes whose corresponding $Z_{tg}$ values are the 200 largest values among all genes.

### Evaluation of the association between the drug and tissue

We evaluated the association between each drug and tissue using network proximity. Suppose for a specific drug j , the module $A_{j}$ consists of its target proteins, which can be obtained from the DrugBank database (Wishart et al., 2018), while the module $B_{t}$ for a specific tissue t contains the tissue-specific proteins. We define the association between drug j and tissue t as the distance between module $A_{j}$ and $B_{t}$ :

$$d_{jt}=\frac{\sum_{a\in A_{j}}\min_{b\in B_{t}}d\left(a,b\right)+\sum_{b\in B_{t}}\min_{a\in A_{j}}d\left(a,b\right)}{\left|A_{j}\right|+\left|B_{t}\right|},$$ (6)

where d(a,b) is the shortest distance between gene a and gene b in the gene–gene regulatory network.

The significance of the association was calculated by the permutation test. Specifically, for each drug j and tissue t pair, we randomly picked up two sets of genes, where the first set contains the same number of genes as the drug–target gene set and the second set contains the same number of genes as the tissue-specific gene set, and then calculated the network distance by Eq. 6. This procedure was conducted 100 times to construct the null distribution of network distance. Suppose the 100 network distances obtained from the permutation procedure are denoted as $d_{jt,1}$ ,..., $d_{jt,100}$ , and we introduced set $D_{jt}$ to contain these 100 distance values. The significance level of the drug–tissue association is denoted as the proportion of distance value in $D_{jt}$ , which is less than $d_{jt}$ .

### One-sided Wilcoxon rank test

We grouped AEs using their System Organ Class (SOC) categories in Medical Dictionary for Regulatory Activities (MedDRA). For a specific SOC category, suppose it contained m AEs in the CEUAFD. We further assumed that for the v -th AE signature, the probabilities for these m AEs were $W_{1v}$ ,…, $W_{mv}$ . To test whether there is a significant association between this SOC category and the v -th AE signature, we reformulated it to test whether the distribution formed by ${\left\{W_{iv}\right\}}_{i=1}^{m}$ is significantly larger than the distribution formed by pooling ${\left\{W_{iv^{\prime }}\right\}}_{i=1}^{m}$ for all $v^{\prime }\neq v$ . This test was performed by the one-sided Wilcoxon rank test.

### Effect size of the association between the SOC category and AE signature

Apart from the one-sided Wilcoxon rank test, we also evaluated the association between SOC categories and AE signatures by calculating effect size (ES). Specifically, for a SOC category containing m AEs, we use $\omega_{v}=$ (*W_1v_* , … ,*W_mv_*) to denote the probabilities for the m AEs in the v -th AE signature, and $\omega_{v}^{c}={\left(W_{1v^{\prime }},\ldots ,W_{mv^{\prime }}\right)}_{v^{\prime }\neq v}$ is a vector containing probabilities for the m AEs in the other three AE signatures. The ES is defined as

$$\text{ES}=\frac{\text{mean}\left(\omega_{v}\right)-\text{mean}\left(\omega_{v}^{c}\right)}{\text{sd}\left(\omega_{v}^{c}\right)}.$$ (7)

### Sparse linear discriminant analysis

We conducted SLDA (Mai et al., 2012) for each pair of drug clusters to pick up discriminant genes. Specifically, for a pair of drug clusters ( $v_{1},v_{2}$ ), suppose there are $n_{1}$ and $n_{2}$ drugs in these two clusters with expression information accessible in the Library of Integrated Network-Based Cellular Signatures (LINCS) A549 database (Subramanian et al., 2017). We assumed that the cluster labels are coded as $y_{j}=-\frac{n}{n_{1}}$ if drug j belongs to cluster $v_{1}$ and $y_{j}=\frac{n}{n_{2}}$ if drug j belongs to cluster $v_{2}$ , where $n_{1}$ and $n_{2}$ are the number of drugs in cluster $v_{1}$ and $v_{2}$ separately, with $n=n_{1}$ + $n_{2}$ . The gene expression profile for drug j was denoted as $x_{j}$ with length p .

The discriminant genes were selected by solving the following *l1* penalized least squares problem:

$$\left(\hat{\beta },{\hat{\beta }}_{0}\right)=\underset{\beta ,\beta_{0}}{\text{argmin}}\left\{n^{-1}\overset{n}{\underset{j=1}{\sum }}{\left(y_{j}-\beta_{0}-x_{j}^{T}\beta \right)}^{2}+\lambda \overset{p}{\underset{k=1}{\sum }}\left|\beta_{k}\right|\right\}.$$ (8)

In practice, we used the R package *TULIP* to solve the abovementioned optimization problem. Because of the limited sample size, cross validation is not reliable in selecting a suitable λ , and we set λ to be the smallest one among the sequence of λ values provided by function *dsda*. Those genes whose $\hat{\beta }$ are non-zero were considered as discriminant genes.

## Results

### AE-signature analysis for approved CEUAFD drugs

The CEUAFD collected from the beginning of the COVID-19 pandemic until September 2021 was downloaded for COVID-19 drug-related AE-signature analysis. We obtained 9,754 reports, corresponding with 15 drugs and 134 unique AE reports after the data-preprocessing procedures (see Supplementary Material). We then applied NMF to the drug-AE count matrix $X\in R^{134\times 15}$ to extract the collection of AE signatures, with each element of X representing the number of patients reporting a specific AE for a particular drug. After a stability-driven model selection procedure (Brunet et al., 2004), we obtained four AE signatures deciphered by NMF, which constituted a sufficient and non-redundant base in depicting the drug-specific AE distributions (see Supplementary Material). We also conducted the clustering of CEUAFD drugs, with drugs in each cluster possessing high abundance fractions on the same AE signature. Some basic information for nine widely used CEUAFD drugs (more than 20 items of AE feedback in the CEUAFD by September 2021) is shown in Table 1. Five out of the nine drugs fall into the second cluster. The two combinational drugs, casirivimab and imdevimab and bamlanivimab and etesevimab, are both enriched with AE signature 3. In contrast, the other two widely used drugs, remdesivir and bamlanivimab, are representative drugs of clusters 1 and 4 separately. Overall, drugs with the most frequent AEs are also representative AEs for each corresponding AE signature within each drug cluster.

**TABLE 1:** Summary of nine CEUAFD drugs. Information on nine widely used CEUAFD drugs (more than 20 items of AE feedback in the CEUAFD by September 2021) is shown, including the number of patients taking one specific drug, the two most frequent AEs, and the cluster to which each drug was assigned.

CEUAFD drugs	#patients	Two most frequent AEs	Clusters
Hydroxychloroquine	71	Electrocardiogram Qt prolonged; hypoglycaemia	2
Remdesivir	4047	Alanine aminotransferase increased; aspartate aminotransferase increased	1
Tocilizumab	71	Alanine aminotransferase increased; aspartate aminotransferase increased	2
Casirivimab and imdevimab	722	Dyspnoea; chills	3
Bamlanivimab	4375	Dyspnoea; pyrexia	4
Baricitinib	123	Lymphocyte count decreased; acute kidney injury	2
Covid-19 convalescent plasma	82	Dyspnoea; chills	2
Bamlanivimab and etesevimab	171	Dyspnoea; pyrexia	3
Vancomycin hydrochloride	23	Acute kidney injury; respiratory failure	2

To give a more general picture of each AE signature, we used the top 20 representative AEs to characterize the rough distributions of four AE signatures (see Supplementary Material) and grouped AEs by their SOC categories in MedDRA. In addition, we performed a one-sided Wilcoxon rank test for every SOC category in each AE signature to determine whether AEs belonging to one SOC category have a high-probability accumulation in some AE-signature distributions.

**FIGURE 2:**
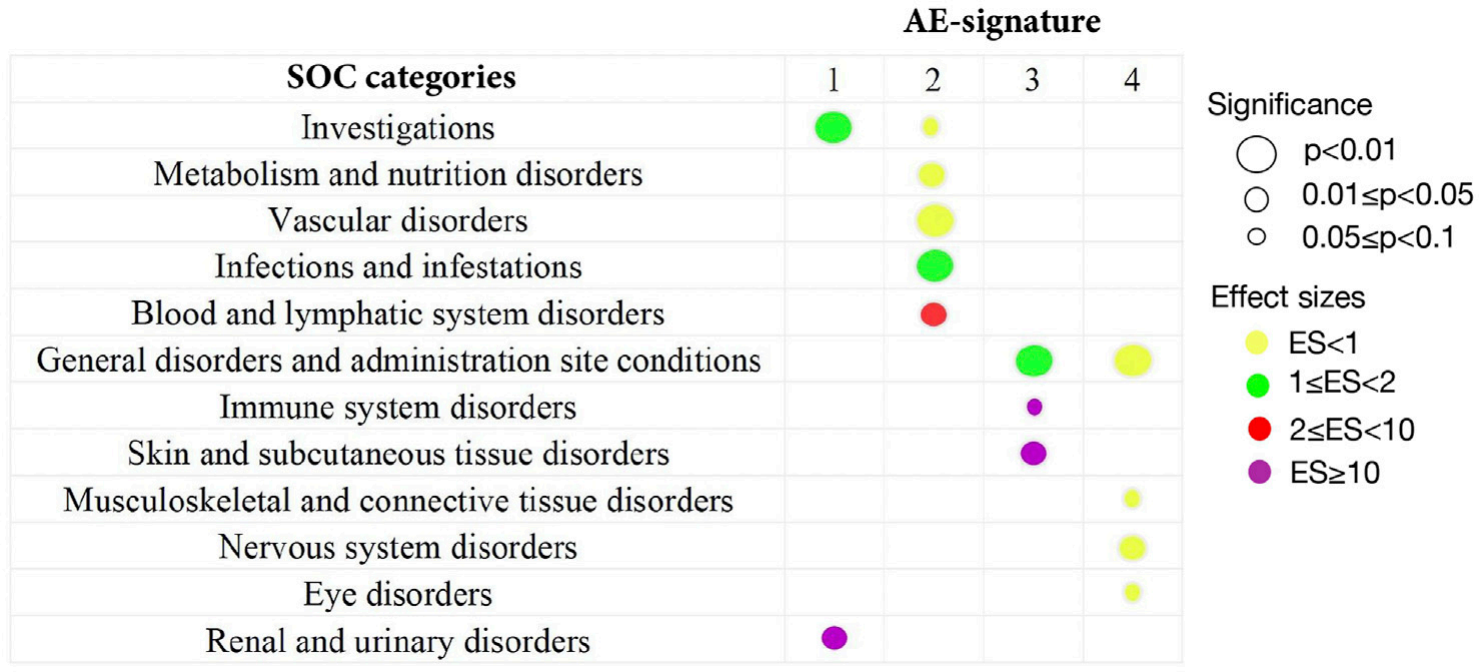
Summary of statistically significant SOC categories returned by the one-sided Wilcoxon rank test. All 134 AEs extracted from the CEUAFD are classified into different SOC categories in MedDRA. By the one-sided Wilcoxon rank test, only significant associations between the SOC category and AE signature are shown. The size of the circles corresponds to different levels of *p* values. The colors represent different effect sizes (ESs) of the association between the SOC category and AE signature.

For the first AE signature, AEs with top probabilities include some liver-related symptoms (alanine aminotransferase increased, aspartate aminotransferase increased, and blood creatinine increased), kidney-related symptoms (acute kidney injury and glomerular filtration rate decreased), and cardiovascular-related symptoms (bradycardia, cardiac arrest, and hypotension) (Supplementary Figure S1). Most of them were classified into SOC categories “investigations” and “renal and urinary disorders.” These two categories correspond to a higher level of AE severity (Figure 2), where the “investigations” category includes some laboratory test indexes, radiologic test indexes, or physical examination indexes. Compared with other drugs, AEs of remdesivir are more abundant in the first AE signature, with more than 90% of AEs attributed to AE signature 1 (Supplementary Figure S2). This finding can be supported by several existing studies which reported the damage to the liver and renal functions of remdesivir users among COVID-19 patients (Perveen et al., 2020; Zampino et al., 2020; Singh and Kamath, 2021). Thus, COVID-19 patients with poor liver, renal, or probably cardiac functions should take remdesivir with caution.

We detected significant aggregation in some kidney-related AEs (acute kidney injury), cardiovascular-related AEs (electrocardiogram QT prolonged, hypotension, and hypoglycemia), and AEs related to the immune system (lymphocyte count decreased, lymphopenia, and leukocytosis) for AE signature 2. This leads to a significant high-probability accumulation of SOC categories “vascular disorder” (*p =* 0.0095) and “blood and lymphatic system disorders” (*p = 0.03*) in AE signature 2 (Figure 2 and Supplementary Figure S3). Hydroxychloroquine, tocilizumab, COVID-19 convalescent plasma, vancomycin hydrochloride, and baricitinib are more enriched with AE signature 2 compared with other drugs (Supplementary Figure S2). Interestingly, tocilizumab and baricitinib are both categorized as “immune modulators” by the FDA when treating COVID-19. Recent research also highlighted that hydroxychloroquine treatment may impair host immunity in response to SARS-CoV-2 (Devarajan and Vaseghi, 2021; Rother et al., 2020), while baricitinib could not be initiated in patients with small lymphocyte count (Praveen et al., 2020). Synthesizing all the abovementioned evidence, we recommend that COVID-19 patients with weak immune systems or vascular disorders should not be prescribed hydroxychloroquine, tocilizumab, or baricitinib.

AE signature 3 and AE signature 4 have many representative AEs in common, such as dyspnoea, pyrexia, hypoxia, oxygen saturation decreased, nausea, vomiting, dizziness, and cough (Supplementary Figures S4, S5). Most of these common representative AEs were classified into the “General disorders and administration site conditions” SOC category (Figure 2). Standing on the SOC category perspective, we can distill more general differences between these two AE signatures—part of the representative AEs in AE signature 3 can be attributed to the “Skin and subcutaneous tissue disorders” SOC category, while AEs belonging to the SOC category “Nervous system disorders” are more abundant in AE signature 4 (Figure 2). Casirivimab and imdevimab and bamlanivimab are the representative drugs of AE signature 3 and 4 separately. Another combinational drug bamlanivimab and etesevimab is also enriched with AE signature 3 (Supplementary Figure S2). Different from the first two AE signatures, the symptoms of the last two AE signatures are relatively mild. This finding is consistent with the FDA’s recommendation that mild-to-moderate COVID-19 in adults and children not admitted to hospital may be treated by bamlanivimab (Mahase, 2020). We also found that reduced oxygen saturation is a common AE brought by both casirivimab and imdevimab and bamlanivimab, which was supported by existing research (Kano et al., 2021). Therefore, casirivimab and imdevimab should be taken carefully by COVID-19 patients with low oxygen saturation.

### AE-signature analysis for drugs in the clinical trial stage

An AE-signature analysis for drugs that are still in the clinical trial stage to fight against COVID-19 was performed. Under the rationale assumption that similar drugs may induce similar side effects (Campillos et al., 2008; Zhang et al., 2017), we predicted their AEs by combining the last-stage similarity network and CEUAFD drugs’ clustering output.

**FIGURE 3:**
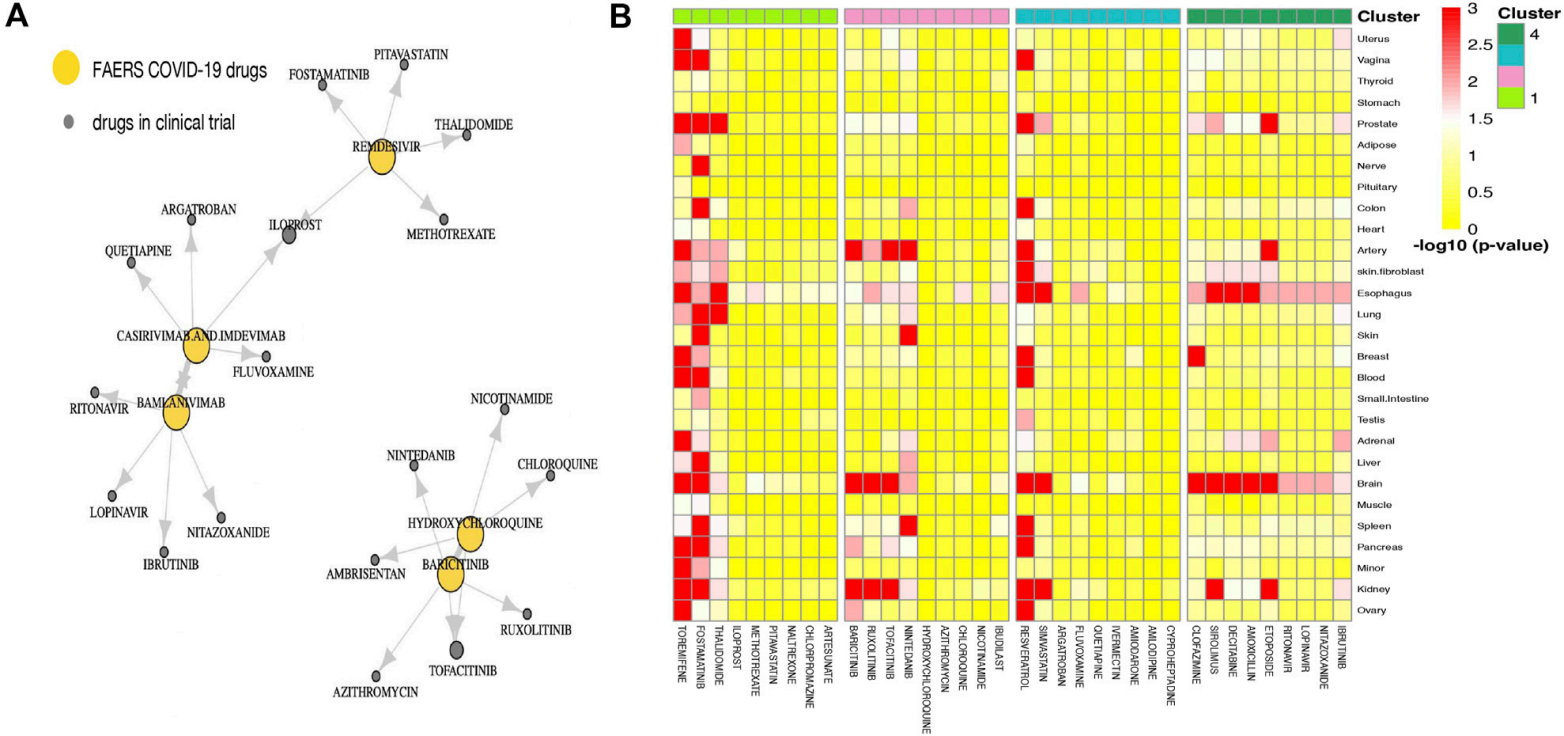
**(A)** Drug–drug similarity network between five widely used CEUAFD drugs and their five nearest neighbor drugs. The arrow pointing from drug A to drug B means drug B is among the five nearest neighbor drugs of drug A. **(B)** Heatmap of the significance of the association between tissues and drugs in four drug clusters. The association for each drug–tissue pair was calculated by network proximity, while the significance of the association was performed using a permutation test. Colors indicate the −log10 (*p* value) of each drug–tissue pair.

Part of the predicted last-stage drug–drug similarity network is presented in Figure 3A, including the five widely used CEUAFD drugs and their five nearest neighbor drugs which were still in the clinical investigation stage. We found that baricitinib and hydroxychloroquine are each other’s five nearest neighbor drugs, which is consistent with the NMF result of the CEUAFD that these two drugs are both representative drugs of AE signature 2. On the other hand, casirivimab and imdevimab and bamlanivimab (both have relatively mild AE feedback from COVID-19 patients) are also among each other’s top five nearest neighbor drugs. Finally, chloroquine, a widely recognized drug with functions similar to hydroxychloroquine, is also among the top five nearest neighbors of hydroxychloroquine. We define drugs from the CEUAFD, i.e., remdesivir, baricitinib, casirivimab and imdevimab, and bamlanivimab, as the representative drugs for the four clusters. With respect to the four drugs, the top 15 nearest neighbor drugs with each are shown in Table 2.

**TABLE 2:** Four drug clusters based on the integrated drug–drug similarity network and NMF outcomes of the CEUAFD. Drugs in bold are from the CEUAFD, and each drug cluster features one specific AE signature.

Cluster 1	Cluster 2	Cluster 3	Cluster 4
**Remdesivir**	**Baricitinib**	**Casirivimab and imdevimab**	**Bamlanivimab**
Fostamatinib	Ruxolitinib	Argatroban	Ritonavir
Iloprost	Tofacitinib	Fluvoxamine	Lopinavir
Thalidomide	Nintedanib	Quetiapine	Nitazoxanide
Methotrexate	**Hydroxychloroquine**	Ivermectin	Ibrutinib
Pitavastatin	Azithromycin	Amiodarone	Clofazimine
Naltrexone	Chloroquine	Amlodipine	Sirolimus
Chlorpromazine	Nicotinamide	Resveratrol	Decitabine
Artesunate	Telmisartan	Cyproheptadine	Amoxicillin
Toremifene	Ibudilast	Simvastatin	Etoposide
Masitinib	Imatinib	Artemisinin	Rivaroxaban
Enzalutamide	Clarithromycin	Ambrisentan	Atorvastatin
Doxycycline	Dexamethasone	Melphalan	Maraviroc
Candesartan	Methylprednisolone	Disulfiram	Leflunomide
Pirfenidone	Prednisone	Dipyridamole	Prazosin

To give a further explanation of the predicted drug clustering result, we applied the network proximity approach to evaluate the association between each drug–tissue pair using the GTEx database. The significance level for each drug–tissue pair is shown in Figure 3B. Compared with the other three drug clusters, more drugs from cluster 1 possess a higher association level in tissues such as the liver, lung, prostate, and blood. There is a strong association between brain tissue and drugs from cluster 4, which gives an indirect reflection that drugs in cluster 4 may induce nervous system disorders. Furthermore, we listed several representative drugs with literature-reported AEs observed in pharmacological and genomic spaces (Table 3). The widely reported AEs showed high-level consistency with the typical AEs in each drug cluster.

**TABLE 3:** Summary of 10 representative COVID-19 drugs in the clinical trial stage. Information on 10representative COVID-19 drugs which are in the clinical trial stage is shown, including DrugBank ID, typical AEs with literature support, and the drug cluster to which each drug belongs.

DrugBank ID	Drug name	AEs reported in the literature	Drug cluster
DB12010	Fostamatinib	Hepatic function impairment, Newland and McDonald (2020)	1
DB01041	Thalidomide	Bradycardia, Thangaraju et al. (2019)	1
DB01088	Iloprost	Acute kidney injury, Uyar et al. (2016); increased GGT, Hsu and Rubin (2005)	1
DB08860	Pitavastatin	Change of glomerular filtration rate, Baik et al. (2019)	1
DB01611	Hydroxychloroquine	Tachycardia and hypotension, Marquardt and Albertson (2001)	2
DB08895	Tofacitinib	Immune system injury, Chen et al. (2017); deep vein thrombosis, Cohen et al. (2020)	2
DB08877	Ruxolitinib	Increased risk of infections and thromboembolic events, Shalabi et al. (2022)	2
DB00176	Fluvoxamine	Dyspnoea, nausea, and headache, Lenze et al. (2020)	3
DB01601	Lopinavir	Acute kidney injury, Binois et al. (2020)	4
DB00507	Nitazoxanide	Gastrointestinal complaints and headache, Walsh et al. (2020)	4

### Molecular mechanisms accounting for different drug clusters

We employed LINCS data with the A549 cell line (Subramanian et al., 2017) and applied SLDA (Mai et al., 2012) to further explore the molecular mechanisms for the four deciphered AE signatures. Our target is to find therapeutic biomarkers presenting distinguishing expression patterns for each pair of drug clusters. Part of the distinguished molecular mechanisms of four AE signatures was reflected by discriminative biomarkers extracted from SLDA.

**FIGURE 4:**
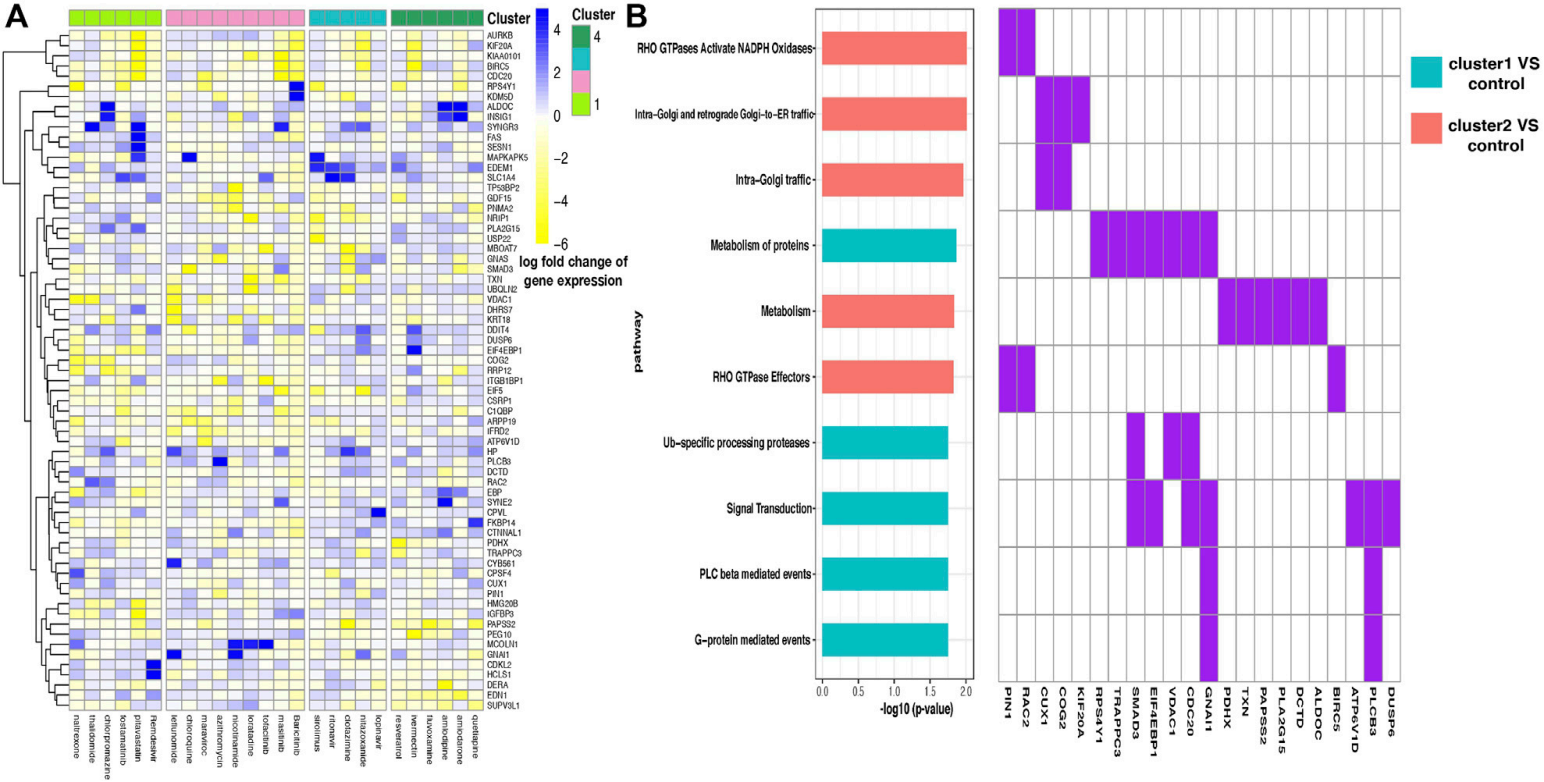
**(A)** Heatmap of the log fold change of gene expression levels for drugs in four clusters. Each row is a gene, and each column is a drug. Drugs are ordered according to their cluster index number. **(B)** Enriched pathways in two discriminant gene sets corresponding to two pairs of drug clusters—drug cluster 1–control pair and 2–control pair. Enrichment analysis was performed in two discriminant gene sets separately, where the two gene sets contain genes showing distinguishing expression patterns between drug cluster 1 and the control group and drug cluster 2 and the control group separately, and the control group was defined as the combination of drug cluster 3 and 4. Ten pathways with the most significant enrichment *p* values are shown, and pathways enriched in different gene sets are filled with different colors (left). Discriminant genes that emerged in the enriched pathways are shown (right).

For every pair of drug clusters, we obtained a discriminative gene set by conducting SLDA. Log fold change of each drug’s expression levels on the selected 67 SLDA genes is shown in Figure 4A. Because representative AEs of drug clusters 3 and 4 are relatively mild and the AEs in clusters 1 and 2 are more severe and characteristic, we merged drug clusters 3 and 4 as the control cluster. SLDA genes showing differential expression patterns in the drug cluster 1–control pair and 2–control pair were collected for further validation. A biological function enrichment analysis was implemented to uncover the possible relationships between enriched pathways and representative differential AEs in the pair of drug clusters (see Supplementary Material).

We found that gene FAS presents highly different expression levels between drug clusters 1 and 2 (Figure 4A). Fas and Fas-ligands are classical members of the TNF receptor and TNF ligand families. The interaction of the receptor with its ligand allows the formation of a death-inducing signaling complex that includes the Fas-associated death domain protein (FADD), caspase 8, and caspase 10. With this mechanism, they play an important role in the regulation of apoptotic processes, including activation-induced cell death, T-cell-induced cytotoxicity, and multiple organ function impairment (Wajant et al., 2003).

By conducting enrichment analysis, the differential genes between drug cluster 1–control pair are enriched in the RHO GTPases Activate NADPH Oxidases pathway, with PIN1 and RAC2 being the pathway input genes (Figure 4B). To our knowledge, RAC2 is an important component of NADPH oxidase (Kim and Dinauer, 2001). Excessive NADPH oxidase-derived ROS production can induce multiple tissue injuries and prolonged inflammatory responses, leading to inflammatory diseases (Belambri et al., 2018).

Different from that mentioned above, the KEGG Reactome analysis showed that a large number of differential genes in the drug cluster 2–control pair flowed to signal transduction pathway (Figure 4B), which is related to many diseases such as cancer, atherosclerosis, and inflammatory diseases. Notably, the SMAD3 gene is selected in this pathway. SMAD3 is a major transcription factor in transforming growth factor- β (TGF- β ) signaling. The TGF- β /Smad-dependent signaling pathway has been shown to be activated in models of myocardial infarction, as well as in multiple pathological processes (Bujak et al., 2007; Peng et al., 2021).

## Discussion

With respect to COVID-19 drug safety, we developed a framework to predict the AE profiles for those drugs which are not in large-scale use. This framework has some advantages from methodological, clinical, and molecular viewpoints.

Some innovative points in our method are worth to be mentioned. The advantages of NMF are two-fold. First, NMF possessed the ability of dimension reduction and drug clustering. Second, by further SOC category enrichment analysis, NMF can capture the subpopulation fragile to each AE signature with a higher precision level. On the other hand, most network integration methods assumed node consistency among different networks. With the rapid development of new drugs, it is hard to fulfill that each drug’s information is available in all data sources. We developed an MDS-based network imputation method, which was achieved by node embedding and building a predictive model employing network-shared drugs. This method can effectively use incomplete information. The imputation is particularly useful when drugs’ information is unable to be presented by low-dimensional vectors (for example, chemical structure or high-dimensional bioassay data), as our imputation model was built in the low-dimensional embedded space. Lastly, we utilized SLDA to seek discriminative genes which may provide molecular-level explanation for different AE signatures among drug clusters. If there is a correspondence between the mechanism of SLDA genes and different AE-signature patterns for each pair of drug clusters, we validate the rationality of our drug clustering results and AE prediction results in some sense. On the other hand, combining symptomatic features with molecular-level features for COVID-19 patients may contribute to more precise therapeutic prescriptions.

Clinically, we presented some important discoveries. Our analysis of the COVID-19 EUA FAERS database showed that remdesivir may bring damage to liver, renal, or probably cardiac functions, which is widely acknowledged with the evolvement of in-depth research (Li et al., 2021). Tocilizumab and baricitinib, which are categorized as “immune modulators” by the FDA, may cause vascular, blood, and lymphatic system disorders in COVID-19 patients. Another important message brought by the CEUAFD is that AE feedbacks from combinational drugs are relatively mild. In addition, by clustering all COVID-19 drugs, we found that among the top five nearest neighbors of Baricitinib, three of them belong to the “tinib” family. Our prediction can be verified by Halimi et al. (2008), who reported that prolongation of the QT interval and heart failure are frequent AEs in patients using tinibs.

From the viewpoint of molecular level, drugs from cluster 1 and control cluster showed differential expression patterns in the RHO GTPases Activate NADPH Oxidases pathway. NADPH oxidase (NOX) is a multimeric transmembrane enzyme complex that uses NADPH as an electron donor to generate superoxide (O2-) and hydrogen peroxide (H2O2) from molecular oxygen. It participates in various biological processes including innate immunity, and biosynthetic processes (Bedard and Krause, 2007). In the pathophysiological process of the liver, NADPH oxidase is expressed functionally in phagocytic and non-phagocytic forms. NOX-derived ROS contributes to various liver diseases caused by alcohol, hepatitis C virus, and toxic bile acids (Paik et al., 2014). In addition to causing liver injury, the impairment of this pathway is also related to renal insufficiency. Abnormally activated NOX in renal microvessels can lead to superoxide production. Oxidative stress in the kidney contributes to renal vascular remodeling and increases preglomerular resistance. Some reports showed that they are key factors in acute and chronic kidney injury (Xu et al., 2020), while for the drug cluster 2–control pair, the differential genes are more relevant to the signal transduction pathway. The relationship between the blockage of the signal transduction pathway and cardiovascular events has been widely reported (Wheeler-Jones, 2005). Previous studies have demonstrated that several kinds of TKIs could lead to grade III or higher QT prolongation, and animal models suggested that this might be caused by the inhibition of PI3K signaling (Schiefer et al., 2018). In addition, the impairment of other signaling pathways is also intimately related to cardiovascular disorders. Mechanistic studies suggested that disturbed TGF- β signaling may also contribute to non-genetic cardiovascular disorders such as atherosclerosis and cardiac fibrosis (Goumans and Ten Dijke, 2018). Reactivation of the WNT signaling pathway has also been observed in many pathologies of cardiac and vascular vessels (Foulquier et al., 2018).

There are also some limitations in our study. Although the CEUAFD provided rich AE feedback for some approved COVID-19 drugs, only 9 drugs were reported by more than 20 patients. This insufficient AE feedback phenomenon may induce inaccuracy in extracting AE signatures. Therefore, the four factorized AE signatures cannot depict AE information for all COVID-19 drugs. Due to the limitations of data accessibility, we only used LINCS data processed on lung tissues to explore molecular-level differences among drug clusters. With the propelling of COVID-19 research, more and more molecular-level data will be released to promote our understanding of the mechanism of AEs returned by different COVID-19 drugs.

## Conclusion

Many existing COVID-19 drugs are still in the clinical trial stage, thus lacking abundant AE feedback. This drug safety analysis tried to solve this important problem by borrowing information from the AE profile of other “old” drugs. Our analysis took the heterogeneous health conditions of COVID-19 patients into consideration and proposed a computational framework to predict AEs for potential COVID-19 drugs. Therefore, the proposed framework jumps out of the current winner-takes-all drug repurposing framework and can provide better precise drug recommendations. We believe that our framework can be generalized to other diseases for precision drug recommendation and drug clustering with reduced time and cost.

## Data Availability

The datasets presented in this study can be found in online repositories. The names of the repository/repositories and accession number(s) can be found in the article/Supplementary Material.
