# Supplementary material for: Adverse effect signature extraction and prediction for drugs treating COVID-19
Source: Front Genet. 2022 Nov 4;13:1019940. doi: 10.3389/fgene.2022.1019940 (PMC9673014; doi:10.3389/fgene.2022.1019940)
Supplement: Supplementary file 1 [file DataSheet1.PDF]

Supplementary Information

Simulation

We designed our simulation under two main scenarios—block-wise and non-block-wise scenarios. We simulated 50 AEs and 100 drugs in each scenario, i.e. the drug-AE matrix $\boldsymbol{X} \in \mathbb{R}^{50\times100}$. Suppose there are 5 underlying AE-signatures, therefore the true factorized matrix $\boldsymbol{W} \in \mathbb{R}^{50\times5}$ and $\boldsymbol{H} \in \mathbb{R}^{5\times100}$. For AE-signatures v (v=1,...,5), suppose the AEs with non-zero probabilities are indexed by set $\mathcal{A}_v$, under the block-wise situation, $\mathcal{A}_v$ are disjoint with each other; while for non-block-wise situation, $\mathcal{A}_v$ can have non-empty intersection with each other.

For matrix $\boldsymbol{H}$, we simulated it by two steps. Firstly, we divided 100 drugs into 5 equal-sized groups, within each group, suppose each drug's abundance fractions on the 5 AE-signatures follows the same Dirichlet distribution. Therefore, we can obtain a simulated drug's intensity matrix $\boldsymbol{H_1}$ with each row summing up to 1. Secondly, we simulated the number of AE reports r_j for each drug j, ranging from 1000 to 3000. By far, we can denote $\boldsymbol{h}_j$, the j-th column of $\boldsymbol{H}$, as $\boldsymbol{h}_j = r_j\boldsymbol{h}_{1j}$, where $\boldsymbol{h}_{1j}$ is the j-th column of $\boldsymbol{H_1}$.

For matrix $\boldsymbol{W}$, each column is a probability distribution corresponding to an AE-signature, we provide the true $\boldsymbol{W}$ under the block-wise and non-block-wise scenarios in the supplementary files.

We simulated the drug-AE matrix as $\boldsymbol{X}$=$\boldsymbol{WH}$ +$\boldsymbol{E}$, where $\boldsymbol{E}$ is the error matrix with poisson noise. NMF was implemented on the noisy matrix $\boldsymbol{X}$.

We run both the two scenarios 100 times to test the performance of NMF in factoring out the underlying AE-signatures and drug's abundance fractions.

NMF has power to extract AE-signatures

The overall picture of AEs for one particular drug is the cumulative combinations of AE-related biological processes returned by patients with different underlying health conditions. It is possible to decipher the signatures of AE-related biological processes by aggregating large amount of drug-AE information. Our objective here resembled the work done by Alexandrov et al. [(Alexandrov et al., 2013)], where the landscape of genome-wide somatic mutations is the cumulative result of all the operative mutational processes. By implementing an NMF framework, Alexandrov et al. [(Alexandrov et al., 2013)] factorized a dictionary of mutation signatures revealing distinct mutagenesis-repair-replication histories of various mutational processes.

In our study, before implementing NMF to the drug-AE matrix given by the COVID-19 EUA FAERS database, we simulated two main scenarios— block-wise or non-block-wise AE-signature patterns— to validate that NMF has a strong power in factoring out both AE-signatures and drugs' abundance fractions on the decomposed AE-signatures. As shown in **Supplementary Figure 6-10**, for a specific AE-signature which can be represented by a probability distribution defined on the whole AE set, the deciphered probability distribution is very close to the true underlying distribution.

NMF performed better in the block-wise scenario than the non-block-wise overall, under the measurement of both distributional distance and robustness. For 100 simulated drugs, we calculated the mean absolute error (MAE) by comparing the factorized drug's abundance fractions with the true abundance fractions on 5 AE-signatures, we can see that an accurate abundance distribution for each drug can be achieved in most simulation runs (**Supplementary Figure 11a**). To further demonstrate that NMF has a strong power to cluster drugs with high accuracy, we performed a sensitivity analysis with respect to the clustering results as shown in **Supplementary Figure 11b**, it can be concluded that both in the block-wise and non-block-wise scenarios, NMF can assign each drug to its deserved cluster accurately.

Datasets

All datasets we used in this study are publicly available, specifically, we used the below datasets:

FAERS general data: FAERS is a self-reporting adverse events system, with substantial information on reports of AEs associated with drugs, and are widely used to study the relationship between drugs and AEs. We downloaded quarterly 10-years FAERS data from 2012 to 2021, which are publicly available at https://fis.fda.gov/extensions/FPD-QDE-FAERS/FPD-QDE-FAERS.html.

COVID-19 emergency use authorization FAERS database (CEUAFD): For drugs under emergency use authorization (EUA) in COVID-19, FDA lunched FAERS Public Dashboard providing selef-reported AEs after taking these drugs. We downloaded data from: https://fis.fda.gov/sense/app/dc0a5af3-51ac-4e10-9791-cdcfa900bd4f/overview

Drug bioassay data: The bioassay results of all analyzed drugs was downloaded from PubChem: https://ftp.ncbi.nlm.nih.gov/pubchem/Bioassay/.

Drug chemical structure data: We used the web-based tool provided by PubChem to calculate the drug-drug similarity from the chemical structure perspective, the website is available in: https://pubchem.ncbi.nlm.nih.gov/score_matrix/score_matrix.cgi, which returned substructure key-based 2D Tanimoto drug-drug similarities.

Gene expression data: For gene expression data of drugs, we applied LINCS database to explore, which can be downloaded from https://www.ncbi.nlm.nih.gov/geo/query/acc.cgi?acc=GSE92742.

For CEUAFD drugs whose gene expression information are not available from LINCS, we obtained their gene expression profiles from NCBI GEO datasets. Specifically, we downloaded gene expression data of Remdesivir from: https://www.ncbi.nlm.nih.gov/geo/query/acc.cgi?acc=GSE154936, gene expression data of Baricitinib was downloaded from: https://www.ncbi.nlm.nih.gov/geo/query/acc.cgi?acc=GSE161664.

GTEx data: We used the RNA-seq data across 28 tissues from GTEx V6 release, which can be download from: https://gtexportal.org/home/datasets.

Data pre-processing of FAERS COVID-19 data

For patients reporting AEs in CEUAFD, in order not to cause confusion in specifying which particular drug had led to AEs, we excluded those COVID-19 patients who took two or more drugs before the occurance of AEs. Furthermore, for each CEUAFD drug, we excluded its very rare AEs whose reporting frequencies accounted for no more than 1% of the overall AEs for this drug. Finally, we obtained 15 drugs and 134 AEs.

Drug-drug similarity score based on FAERS, bioassay and chemical structure data

For FAERS general data, the AE similarity of two drugs was measured using a Pearson correlation of the AE profiles.

For bioassay data, we first reformulated the data to a binary variable (i.e., positive or negative). Then we used Jaccard index to represent similarity of drug j_1 and drug j_2 , i.e.,

$$S_{\mathrm{assay}}(j_1, j_2) = \frac{|A_{\mathrm{j}_1} \cap A_{\mathrm{j}_2}|}{|A_{\mathrm{j}_1}| + |A_{\mathrm{j}_1}| - |A_{\mathrm{j}_1} \cap A_{\mathrm{j}_2}|}$$

Where A_{j_1} and A_{j_2} are the number of assays applied to drug j_1 and drug j_2 separately, while $|A_{\mathrm{j}_1} \cap A_{\mathrm{j}_2}|$ is the number of shared positive assays between the two drugs.

For calculating the drug-drug similarity based on chemical structure data, as aforementioned, we applied the PubChem web-based tool to calculate the 2D Tanimoto drug-drug similarity.

Drug-drug similarity score based on gene expression data

For gene expression data, we conducted mainly two steps to calculate drug-drug similarity score.

The first step mainly focused on the LINCS database, which provides changes in gene expression when cells are exposed to a variety of compounds (Subramanian et al., 2017). We extracted expression profiles on 12328 genes for 102 COVID-19 drugs processed in A549 cell line. The similarity score between each pair of drugs was calculated by connectivity score (Lamb et al., 2006).

In the second step, we considered COVID-19 drugs whose expression information are not available in LINCS database but are accessible in GEO database, such as Remdesivir and Baricitinib. Besides calculating drug-drug similarity score among these drugs based on information offered in GEO database, we also aimed to calculate similarity score between drugs in the second step and drugs in the first step.

Due to the heterogeneous sequencing platforms, different cell lines of LINCS data and gene expression data extracted from GEO database, we also adopted the connectivity score put forward by Lamb et al. (Lamb et al., 2006), which employed a nonparametric rank-based pattern-matching strategy based on the Kolmogoriv-Smirnov statistic. The connectivity score only utilized the rank information based on the log2 fold change of after-treatment and before-treatment

gene expression data, filtering some underlying nuisance effects brought by different platforms or tissue origins (Lamb et al., 2006).

We downloaded gene expression data of Remdesivir from GEO with Accession number GSE154936, which provided gene expression profiles processed with four different cell lines and two different processing times. The expression correlation outcomes in (Akinci et al., 2020) showed that, while Remdesivir was processed with the same cell line but different processing times, the correlation of gene expression profiles among different processing times are relatively high, this is also the case for DMSO (control). So within each cell line, we merged the gene expression data with different processing times by taking the average of read counts for each gene. Furthermore, in each cell line i $(i = 1, \ldots, 4)$, we calculated the connectivity scores between Remdesivir and all other COVID-19 drugs whose gene expression data are available in LINCS database, forming a vector CL_i containing connectivity scores. Meanwhile, we computed an integrated drug-drug similarity network $\boldsymbol{S_3}$ utilizing other three databases by applying similarity network fusion (SNF, (Wang et al., 2014). We extracted Remdesivir's nearest 20 drugs from $\boldsymbol{S_3}$, the connectivity scores between Remdesivir and these nearest neighbour drugs in different cell lines are shown in **Supplementary Table 1**. Based on the principle that there should be a rough consistency in selecting similar drugs with Remdesivir according to $\boldsymbol{S_3}$ and CL_i for some specific i, we chose merged gene expression profile in HepG2 cell line.

We ultilized GEO dataset with Accession number GSE161664 to extract gene expression information of Baricitinib, we merged the replicates by averaging the before-treatment and after-treatment data respectively.

Last stage drug-drug similarity network construction

After conducting MDS-based network imputation procedure, we can obtain four dimension-consistent networks. We then applied SNF (Wang et al., 2014) to integrate these four similarity networks to construct an informative last-stage network, compared with traditional nonlinear integration method (Huang et al., 2021), SNF is more robust to extreme similarity scores. We denote the integrated similarity network as $\boldsymbol{S}$ with element $S(j, j')$ representing the integrated similarity between drug j and drug j'.

Choice of the number of AE-signatures

To determine the number of AE-signatures, i.e. the value of k, we combined the stability-driven model selection method as that in (Brunet et al., 2004) and the measure of residual sum of squares (RSS). The cophenetic correlation reflecting the stability level and RSS values under different choice of k are depicted in **Supplementary Figure 12**, we set k=4 considering the combined results.

Normalization of factorized matrix $\boldsymbol{W}$ and $\boldsymbol{H}$

For ease of interpretation, we further normalized $\boldsymbol{W}$ to make the summation of each column to be 1. Thus, each AE-signature is represented by a discrete probability defined on the p unique AEs. Withous loss of generalization, we assumed that $\boldsymbol{W}$

has been normalized when showing our main results.

To account for different usage scales of drugs, we further rewrote $\boldsymbol{h}_j$, i.e., the j-th column of $\boldsymbol{H}$, as $\boldsymbol{h}_j^T$= $(h_{1j}^*,h_{2j}^*,...,h_{kj}^*) \times \sum_{m=1}^{k} h_{mj}$ with $h_{vj}^* = \frac{h_{vj}}{\sum_{m=1}^{k} h_{mj}}$, and each h_{vj}^* is the proportion of patients who shows the v-th AE-signature among all patients taking drug j for $v = 1,...,k$ and $j = 1,...,n$. For convenience, we also refer to $\boldsymbol{h}_j^T$ as abundance fractions on the k factorized AE-signatures for drug j. As that in $\boldsymbol{W}$, we also assumed $\boldsymbol{H}$ has been normalized by column in the main results.

Choice of top R representative AEs for each AE-signature

For the ease of biological interpretation for AE-signature, besides using SOC categories, we also summarized the v-th AE-signature using several representative AEs with large probability, i.e. we selected R AEs corresponding with top R largest numbers in $\boldsymbol{w}_v$, where $\boldsymbol{w}_v$ represents the v-th column of $\boldsymbol{W}$.

Before presenting the principles in the choice of R, we introdue some symbols first. For drug j, if $v^* = \underset{v=1,...,k}{\text{argmax}}\, h_{vj}$, we clustered drug j into cluster v^*. Suppose $\boldsymbol{C} \in \mathbb{R}^{n\times n}$ is the drugs' clustering matrix based on $\boldsymbol{H}$, C_{j_1,j_2}=1 if drug j_1 and drug j_2 belong to the same cluster, C_{j_1,j_2}=0 otherwise. Furthermore, we denote $\mathcal{E}_v^R$ as the set containing indexes of AEs with top R largest values in $\boldsymbol{w}_v$, suppose $\mathcal{E}^R = \bigcup_v \mathcal{E}_v^R$ and $\boldsymbol{W}_{\mathcal{E}^R} \in \mathbb{R}^{\mathcal{E}^R \times k}$ is a sub-matrix of $\boldsymbol{W}$, i.e., suppose the i-th row of $\boldsymbol{W}$ is denoted as $\widetilde{w}_i$, only those $\widetilde{w}_i$ with AE index $i \in \mathcal{E}^R$ can constitute the rows of $\boldsymbol{W}_{\mathcal{E}^R}$. Similarly, ultilizing the same set $\mathcal{E}^R$, we define a new sub-matrix of $\boldsymbol{X}$ as $\boldsymbol{X}_{\mathcal{E}^R}$. If we denote $\boldsymbol{H}_R = \boldsymbol{W}_{\mathcal{E}^R}^{g}\boldsymbol{X}_{\mathcal{E}^R}$, where $\boldsymbol{W}_{\mathcal{E}^R}^{g}$ is the generalized inverse of $\boldsymbol{W}_{\mathcal{E}^R}$, we can also get a clustering matrix $\boldsymbol{C}_R$ based on $\boldsymbol{H}_R$.

The rationale in selecting R can be summarized as follows. The columns of $\boldsymbol{W}$ forms the base of AEs, the j-th column of $\boldsymbol{H}$ can be regarded as the coordinates of drug j after projection into the AE space. For each choice of $R < n$, the columns of $\boldsymbol{W}_{\mathcal{E}^R}$ forms a new low-dimensional base of AEs, we regard it as an informative base in the sense that the drug's clustering result based on $\boldsymbol{H}_R$ should not deviate too much from the clustering result based on the original $\boldsymbol{H}$, in other words, $||\boldsymbol{C}_R - \boldsymbol{C}||_F$should be lower than a given acceptable threshold. The values of $||\boldsymbol{C}_R - \boldsymbol{C}||_F$ under different choices of R are shown in **Supplementary Figure 13**, we set R=20 in the FAERS COVID-19 database.

Precision drug recommendation for the treatment of COVID-19

Based on the last-stage drug-drug similarity network and AE-signature analysis of CEUAFD, we can make precise medical suggestions for the drugs which are still in clinical trial stage but not approved yet in treating COVID-19. Specifically, denote the

drug nodes in the laste-stage drug-drug similarity network as $\mathcal{D}_1$ with $\mathcal{D}_1$=N, and the drug set in the NMF analysis as $\mathcal{D}_2$ with $\mathcal{D}_2$=n. So $\mathcal{D}_1 \supset \mathcal{D}_2$ and N>n. For each drug in $\mathcal{D}_2$, we extracted those drugs in $\mathcal{D}_1$ who had high similarity with it. Based on the clustering procedure of CEUAFD drugs, we can obtain k groups of drugs and each group contains at least one drug in $\mathcal{D}_2$ whose AE-signature information is accessible based on the NMF procedure.

Suppose from the NMF procedure regarding the CEUAFD, we had identified a sub-population $P_v$who had a tendency to result in the representative AEs of the v-th AE-signature after taking drug $j' \in \mathcal{D}_2$. If we define a set $\mathcal{G}$ containing drugs whose similarity score with drug j' is among the 10 largest values in the j'-th row of last-stage similarity matrix $\boldsymbol{S}$, we can give precise recommendations to sub-population P_v that they might be of high risk facing the v-th AE-signature after taking drugs $j \in \mathcal{G}$.

Gene set enrichment analysis

For each discriminative gene set, we conducted enrichment analysis to pick up pathways showing discriminative patterns for each pair of drug clusters. The gene set enrichment analysis was implemented in KOBAS-i (Bu et al., 2021), where the enrichment score is an integration of seven functional class scoring (FCS) methods and two pathway topology (PT) methods based on a novel machine learning approach (Ai and Kong, 2018).

Sensitivity analysis of network imputation

We conducted a sensitivity analysis with respect to different choices of k_f and k_a in the imputation process, where k_f and k_a are dimensions of the Euclidean space where drugs are embedded in after multidimensional scaling (MDS). If the four second-stage networks do not vary much with different choices of k_f and k_a, the robustness of the MDS-based imputation method can be guaranteed.

If $\lambda_{f,1} \geq \ldots \geq \lambda_{f,n_f} \geq 0$ and $\lambda_{a,1} \geq \ldots \geq \lambda_{a,n_a} \geq 0$ are eigenvalues of the double-centering matrix $\boldsymbol{C}_f$ and $\boldsymbol{C}_a$ respectively, the plots for the eigenvalues are shown in **Supplementary Figure 14**, which can help us determine the rough range of k_f and k_a (here we use $\boldsymbol{C}_a$ to represent the double-centering matrix with respect to bioassay, chemical structure or gene expression information, $\boldsymbol{C}_f$ represents the double-centering matrix with respect to FAERS database).

From the four eigenvalue plots as shown in **Supplementary Figure 14**, we determined to let k_f take values 5 and 6, let k_a take values from the set (30, 32, 34, 36, 38, 40, 42, 44, 46, 48, 50), leading to 11*2=22 different choices.

For each choice, we conducted the MDS-based imputation and similarity network fusion (SNF) procedures. The sensitivity is measured by accessing whether the top 20 nearest drugs of five representative drugs (Remdesivir, Hydroxychloroquine, Baricitinib, Casirivimab&Imdevimab and Bamlanivimab) are varied largely with different choices of k_f and k_a. Concretely, we take Remdesivir as an example. For the i-th choice of k_f and k_a, suppose the top 20 nearest drugs of Remdesivir

constitute a set R_i. For overall 22 choices, suppose the intersection set of these 22 R_i forms a new set R, i.e. $R = \bigcap_{i=1}^{22} R_i$, if $|R|$ (the cardinality of set R) is not far away from 20, we can claim that our MDS-based imputation method is robust.

After calculation, we reached the outcome that $|R|$=19, 20, 20, 13, 15 for drug Remdesivir, Hydroxychloroquine, Baricitinib, Casirivimab&Imdevimab and Bamlanivimab separately, indicating a high sensitivity/robustness level.

Supplementary tables and figures

Supplementary Table 1 Connectivity score between Remdesivir and its nearest 20 drugs from S_3 in four different cell lines. S_3 is the integrated drug-drug similarity network by utilizing FAERS, bioassay and chemical structure database. In each cell line, the connectivity score between Remdesivir and its nearest 20 drugs from S_3 was calculated, aiming to determine the expression data of Remdesivir in which cell line to use.

	PC3_HCT116_Rem_8h_24h	PC3_HepG2_Rem_8h_24h	PC3_HT29_Rem_8h_24h	PC3_PLC.PRF.5.Cas9NG.mChe_Rem_8h_24h
Fostamatinib	-0.000964917	0.998331504	0.964360308	0.005463927
Iloprost	-0.006318688	0.996745918	0.968893068	0.011635822
Ibrutinib	-0.001099101	0.993653744	-0.022463062	0.972198331
Artesunate	0.002313829	0.991729637	0.992668755	0.003648939
Itraconazole	-0.006990085	0.99235728	0.995154666	1
Prazosin	-0.00455152	0.992609084	-0.014489777	-0.979464684
Argatroban	0.000714885	0.990738223	0.956993143	0.014532732
Nitazoxanide	0.00637448	0.991550971	-0.019121994	0.007531368
Clofazimine	0.007587078	0.993442309	-0.035099769	0.017543364
Artemisinin	-0.01773267	0.000792716	-0.020959953	0.022843041
Dipyridamole	-0.00252768	0.002823837	0.940968093	-0.986060563
Pioglitazone	-0.00046435	0.013704228	-0.01707261	0.967173131
Disulfiram	0.002122493	-0.010117017	-0.035783371	-0.98354479
Rivaroxaban	0.991346731	-0.002832078	-0.011909846	0.017672902
Pentoxifylline	-0.006571211	0.989993028	-0.075751296	-0.986220012
Lopinavir	0.996391325	0.988703377	0.907708583	0.016958529
Cyproheptadine	0.985360279	0.988967957	0.979832466	0.020198584
Ritonavir	-0.98847318	-0.987782803	-0.01724996	0.979776462
Celecoxib	0.982577725	0.99299593	-0.012284588	0.987359957
Mefloquine	-0.996761082	0.002411413	-0.985094021	0.990332425

Supplementary Table 2 Clustering result for all studied COVID-19 drugs. All studied COVID-19 drugs are clustered into four clusters based on the last-stage network and NMF results of CEUAFD drugs, with each cluster featuring one AE-signature. Drugs in bold are from CEUAFD.

Cluster 1	Cluster 2	Cluster 3	Cluster 4
**Remdesivir**	**Baricitinib**	**Casirivimab&Imdevimab**	**Bamlanivimab**
Fostamatinib	Ruxolitinib	Argatroban	Ritonavir
Iloprost	Tofacitinib	Fluvoxamine	Lopinavir
Thalidomide	Nintedanib	Quetiapine	Nitazoxanide
Methotrexate	**Hydroxychloroquine**	Ivermectin	Ibrutinib
Pitavastatin	Azithromycin	Amiodarone	Clofazimine
Naltrexone	Chloroquine	Amlodipine	Sirolimus
Chlorpromazine	Nicotinamide	Resveratrol	Decitabine
Artesunate	Telmisartan	Cyproheptadine	Amoxicillin
Toremifene	Ibudilast	Simvastatin	Etoposide
Masitinib	Imatinib	Artemisinin	Rivaroxaban
Enzalutamide	Clarithromycin	Ambrisentan	Atorvastatin
Doxycycline	Dexamethasone	Melphalan	Maraviroc
Candesartan	Methylprednisolone	Disulfiram	Leflunomide
Pirfenidone	Prednisone	Dipyridamole	Prazosin
Losartan		Fenofibrate	Itraconazole
		Colchicine	Celecoxib
		Loratadine	Pentoxifylline
		Diphenhydramine	Pioglitazone
		Verapamil	Sildenafil
		Propofol	Fluoxetine
		Quercetin	Tacrolimus
		Rosuvastatin	Hesperidin
		Midazolam	Famotidine
		Budesonide	Paracetamol
		Clopidogrel	Sitagliptin
		Mefloquine	Melatonin
		Modafinil	
		Trimetazidine	
		Montelukast	
		Ibuprofen	
		Metformin	
		Lidocaine	
		Amantadine	

		Tramadol	
		Fluticasone	
		Hydrocortisone	
		Prednisolone	

Supplementary Figure 1 Barplots of top 20 representative AEs for the first extracted AE-signature. AEs with top 20 probabilities in the first AE-signature are shown. Y-axis denotes the corresponding probability value for each AE.

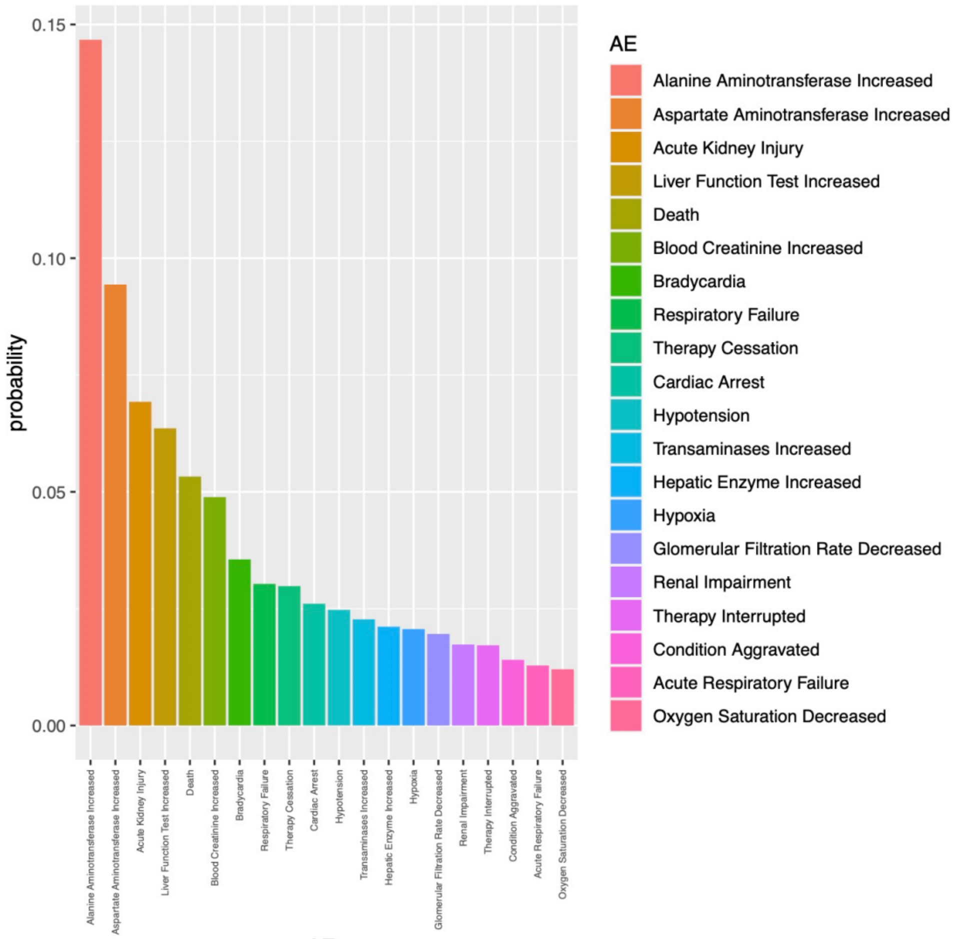

Supplementary Figure 2 Abundance fractions on the four AE-signatures for nine widely-used CEUAFD drugs. For nine widely-used CEUAFD drugs, their abundance fractions on the four extracted AE-signatures are shown, where the abundance fractions are based on the matrix $\boldsymbol{H}$ obtained in the NMF procedure.

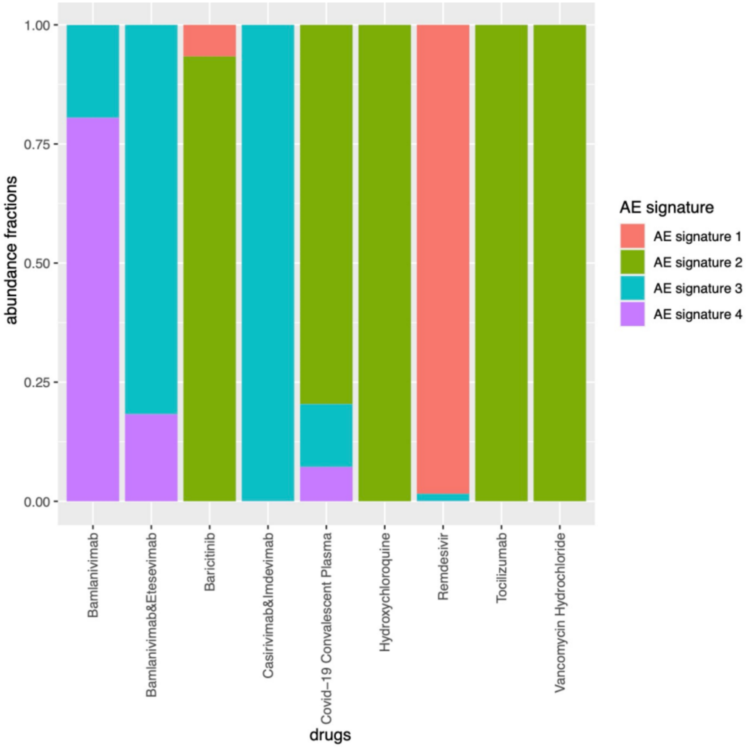

Supplementary Figure 3 Barplots of top 20 representative AEs for the second extracted AE-signature. AEs with top 20 probabilities in the second AE-signature are shown. Y-axis denotes the corresponding probability value for each AE.

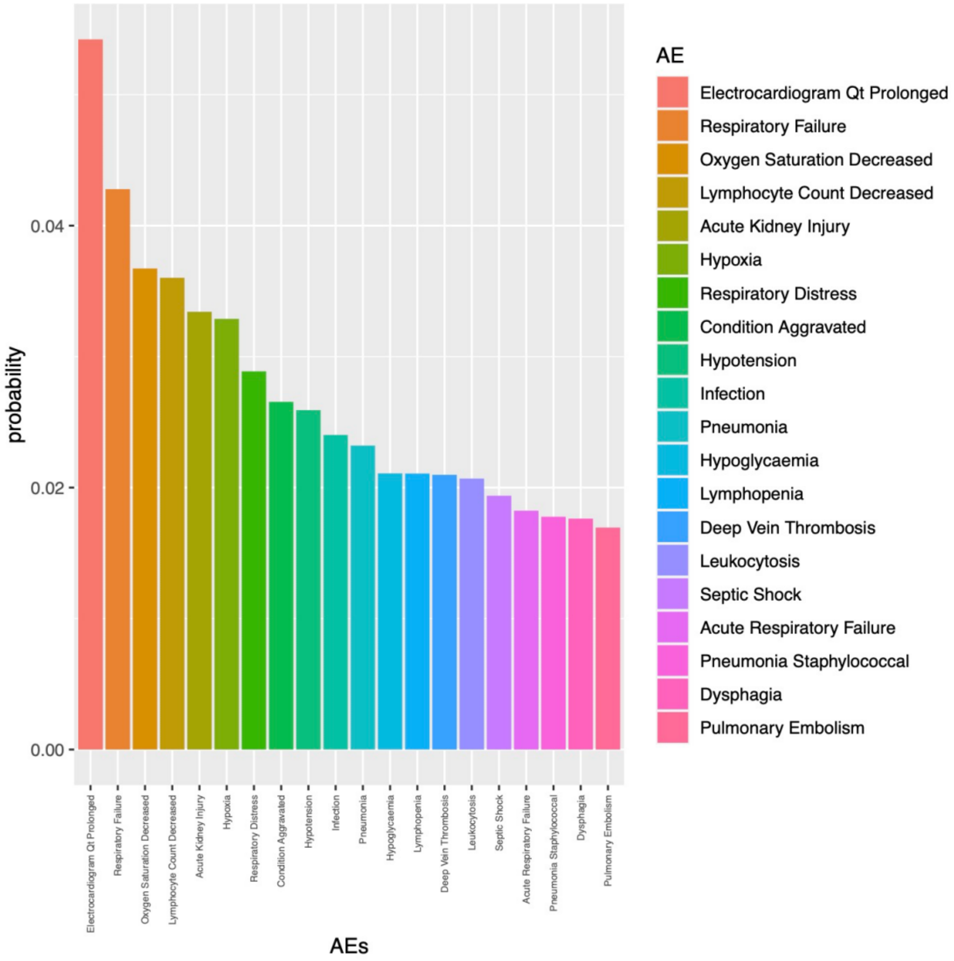

Supplementary Figure 4 Barplots of top 20 representative AEs for the third extracted AE-signature. AEs with top 20 probabilities in the third AE-signature are shown. Y-axis denotes the corresponding probability value for each AE.

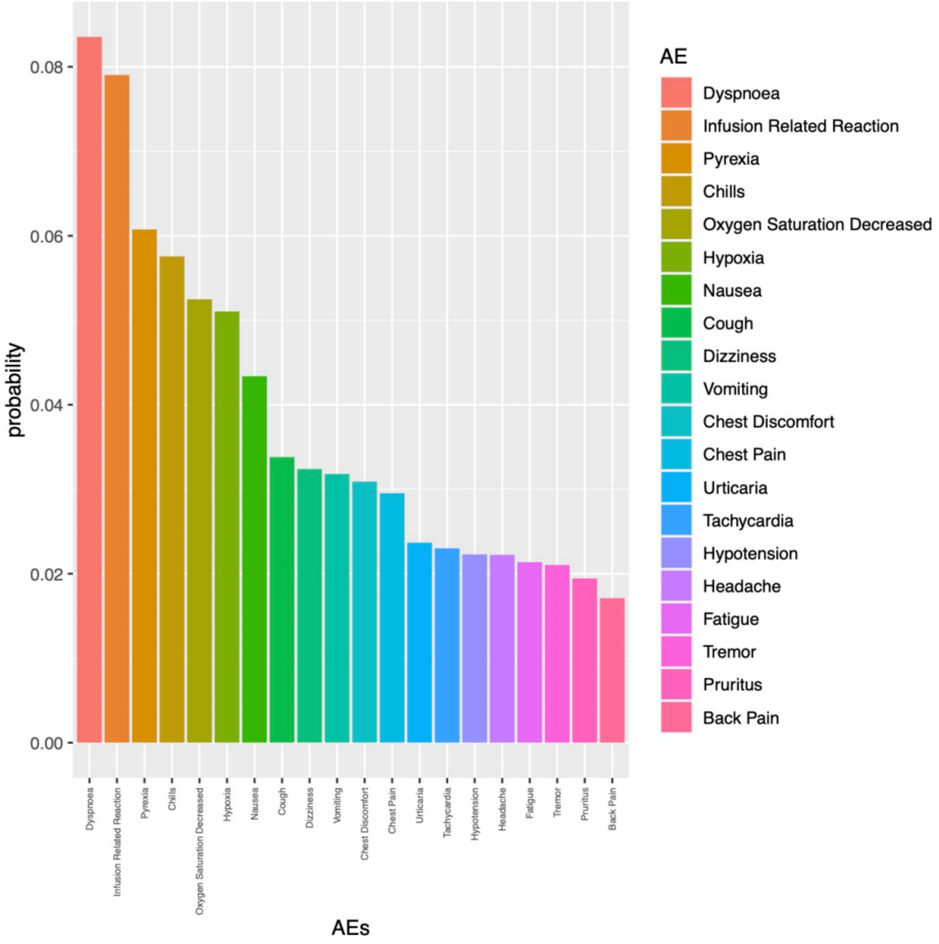

Supplementary Figure 5 Barplots of top 20 representative AEs for the forth extracted AE-signature. AEs with top 20 probabilities in the forth AE-signature are shown. Y-axis denotes the corresponding probability value for each AE.

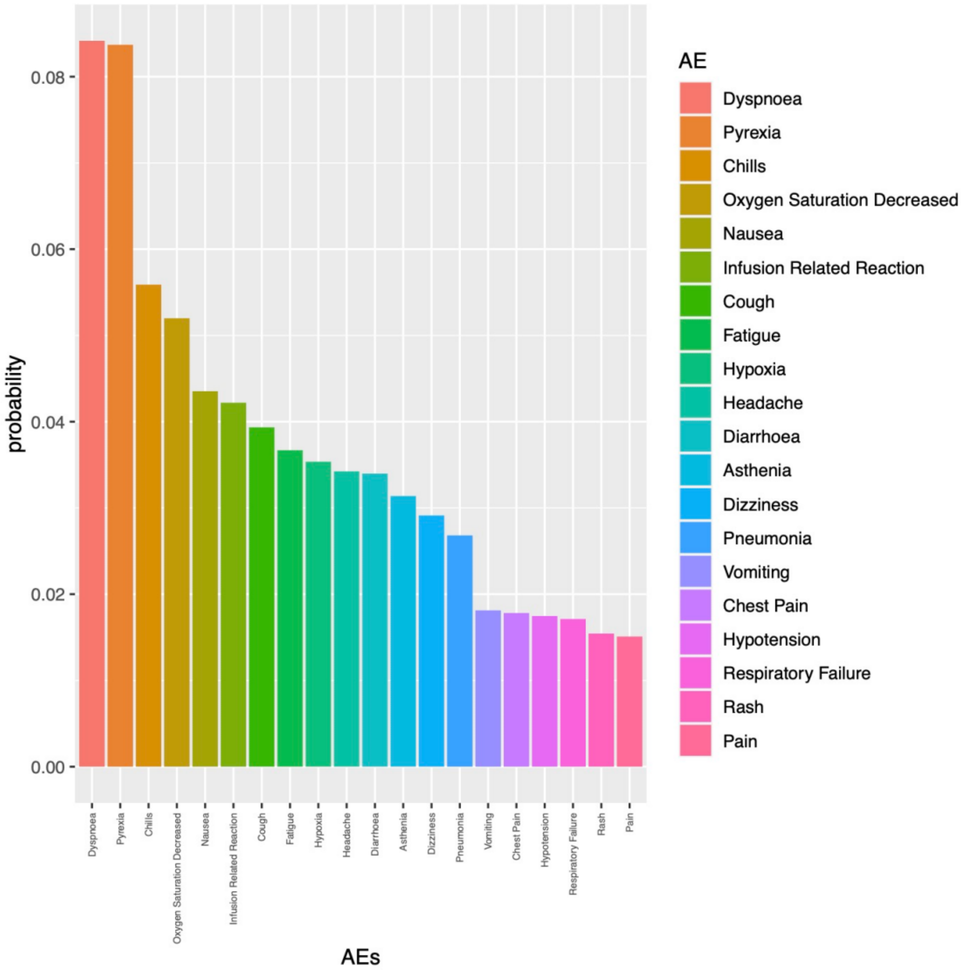

Supplementary Figure 6 Simulation performance in extracting the first AE-signature under the block-wise scenario and non-block-wise scenario. a, the block-wise scenario, x-axis is the index of 50 AEs, y-axis is the probability corresponding to each AE. Purple diamonds are the true probability values for the first AE-signature, red boxplots correspond to the extracted probabilities obtained from NMF for the first AE-signature by simulation under 100 run times. **b**, the non-block-wise scenario, x-axis is the index of 50 AEs, y-axis is the probability corresponding to each AE. Purple diamonds are the true probability values for the first AE-signature, blue boxplots correspond to the extracted probabilities obtained from NMF for the first AE-signature by simulation under 100 run times.

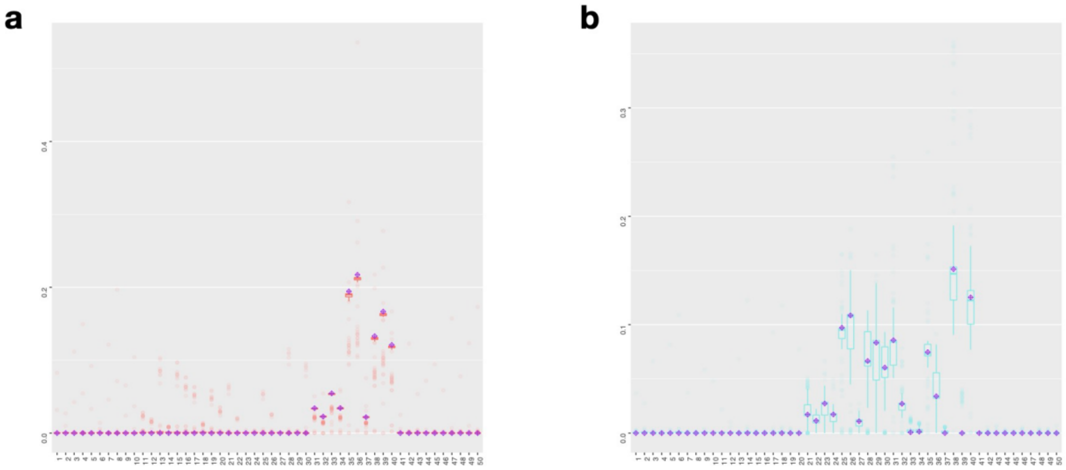

Supplementary Figure 7 Simulation performance in extracting AE-signatures 2 under the block-wise scenario and non-block-wise scenario. The same as that in Supplementary Fig. 6.

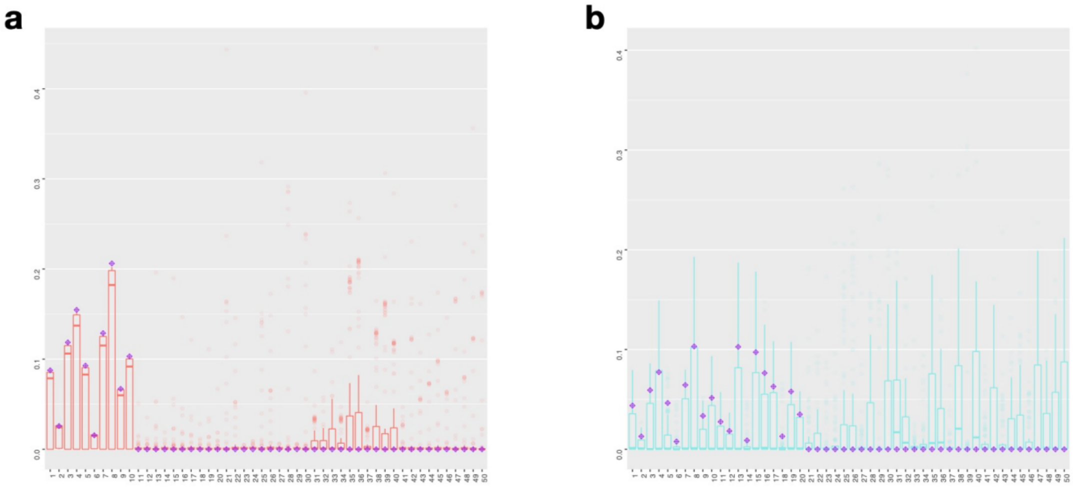

Supplementary Figure 8 Simulation performance in extracting AE-signatures 3 under the block-wise scenario and non-block-wise scenario. The same as that in Supplementary Fig. 6.

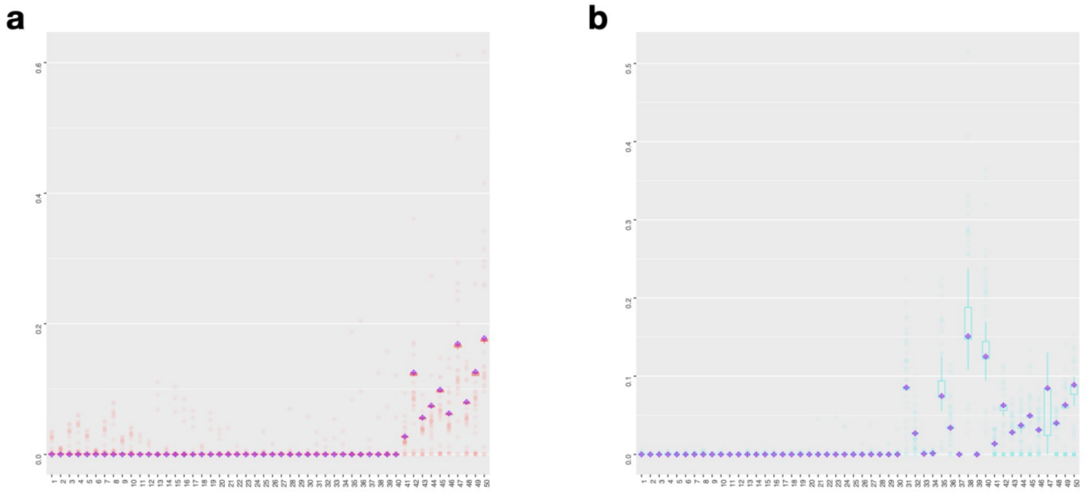

Supplementary Figure 9 Simulation performance in extracting AE-signatures 4 under the block-wise scenario and non-block-wise scenario. The same as that in Supplementary Fig. 6.

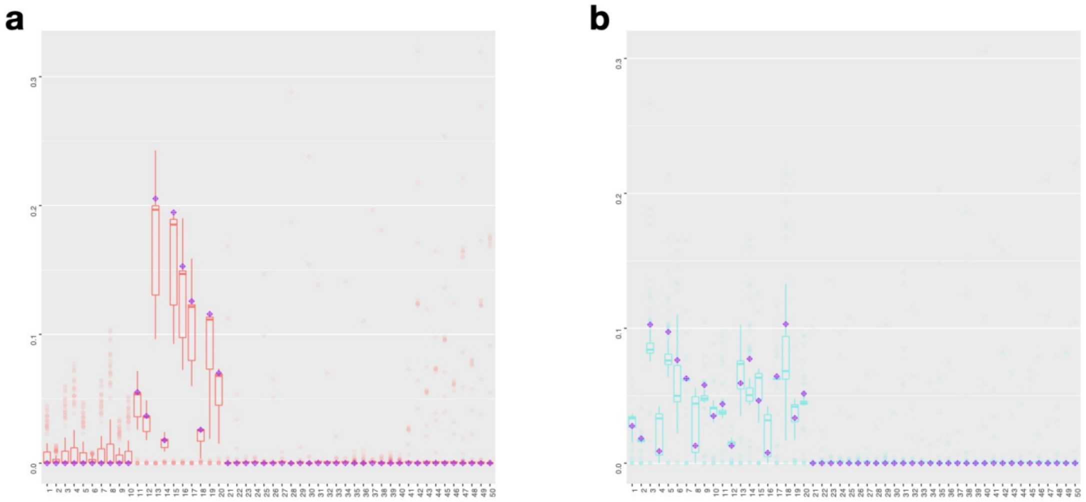

Supplementary Figure 10 Simulation performance in extracting AE-signatures 5 under the block-wise scenario and non-block-wise scenario. The same as that in Supplementary Fig. 6.

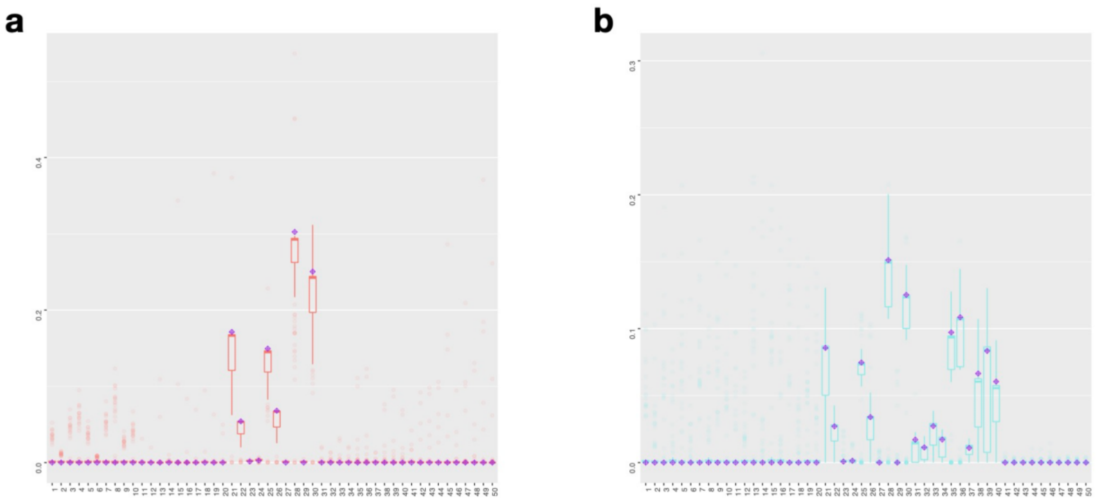

Supplementary Figure 11 a, Simulation performance in extracting abundance fractions for 100 simulated drugs. X-axis is the index of 100 simulated drugs, y-axis is the mean absolute error between the extracted abundance fractions and the true abundance fractions on the 5 AE-signatures for each drug, the boxplots correspond to the mean absolute error for 100 simulation runs. **b,** Sensitivity results in describing the power of NMF for clustering of drugs under the block-wise and non-block-wise scenarios. Each dot represents the proportion of drugs which are clustered into the correct cluster under each run of simulation. Simulation was conducted 100 times.

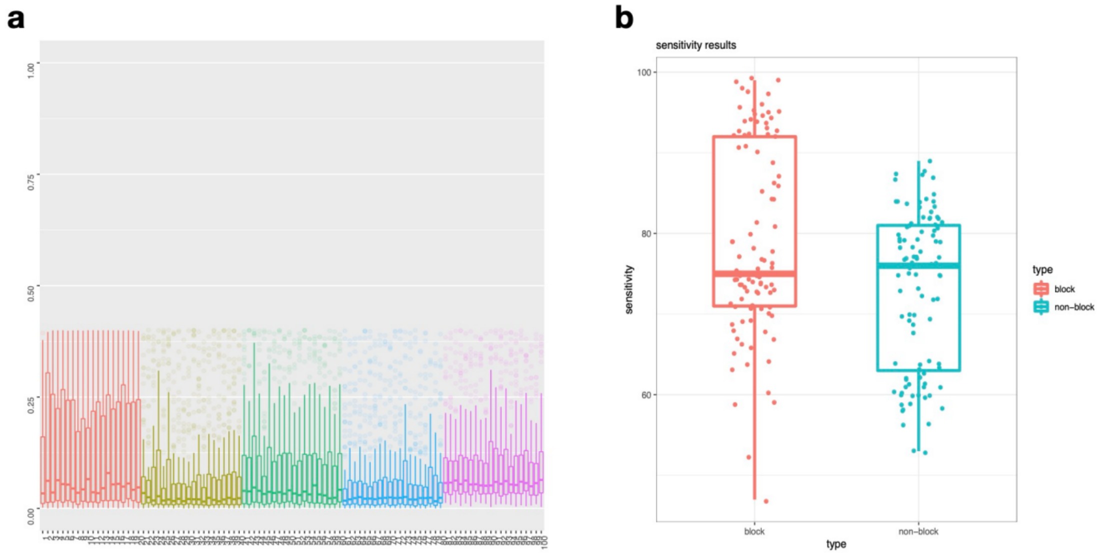

Supplementary Figure 12 Determine the number of AE-signatures. a, Cophenetic correlation (y-axis) reflecting the stability level under different number of AE-signatures (x-axis). **b**, Residual sum of squares (y-axis) under different number of AE-signatures (x-axis).

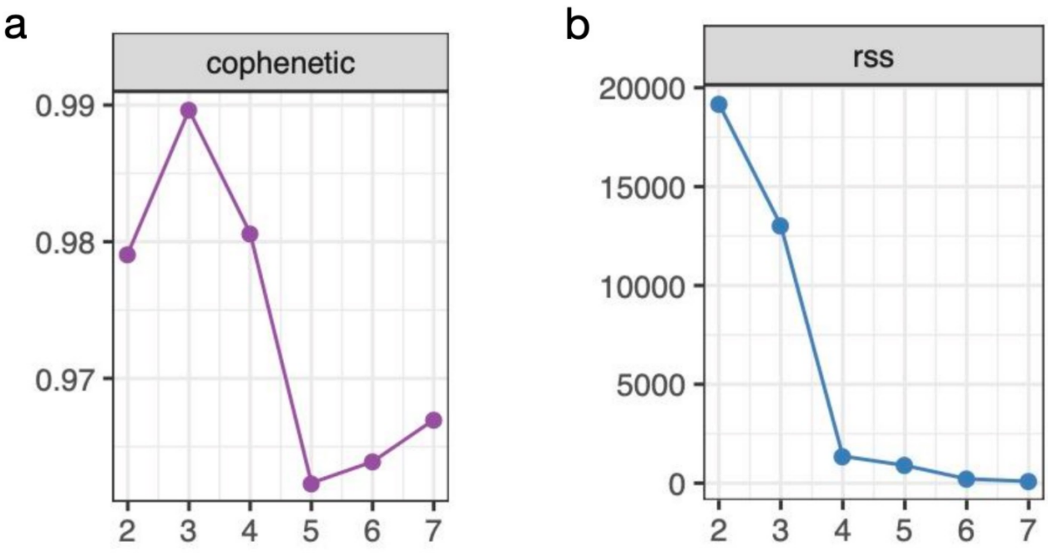

Supplementary Figure 13 Determine the number of representative AEs. $||\boldsymbol{C}_R - \boldsymbol{C}||_F$ values (y-axis) under different numbers of representative AEs (x-axis).

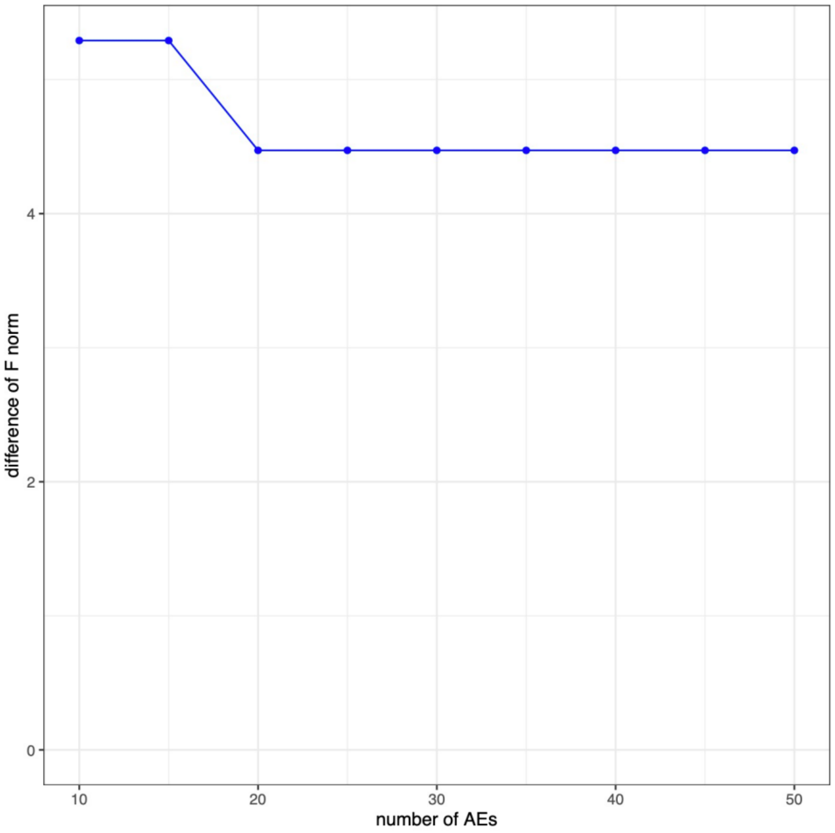

Supplementary Figure 14 Eigenvalue plots of four double-centering matrix with respect to FAERS, bioassay, chemical structure and gene expression datasets.

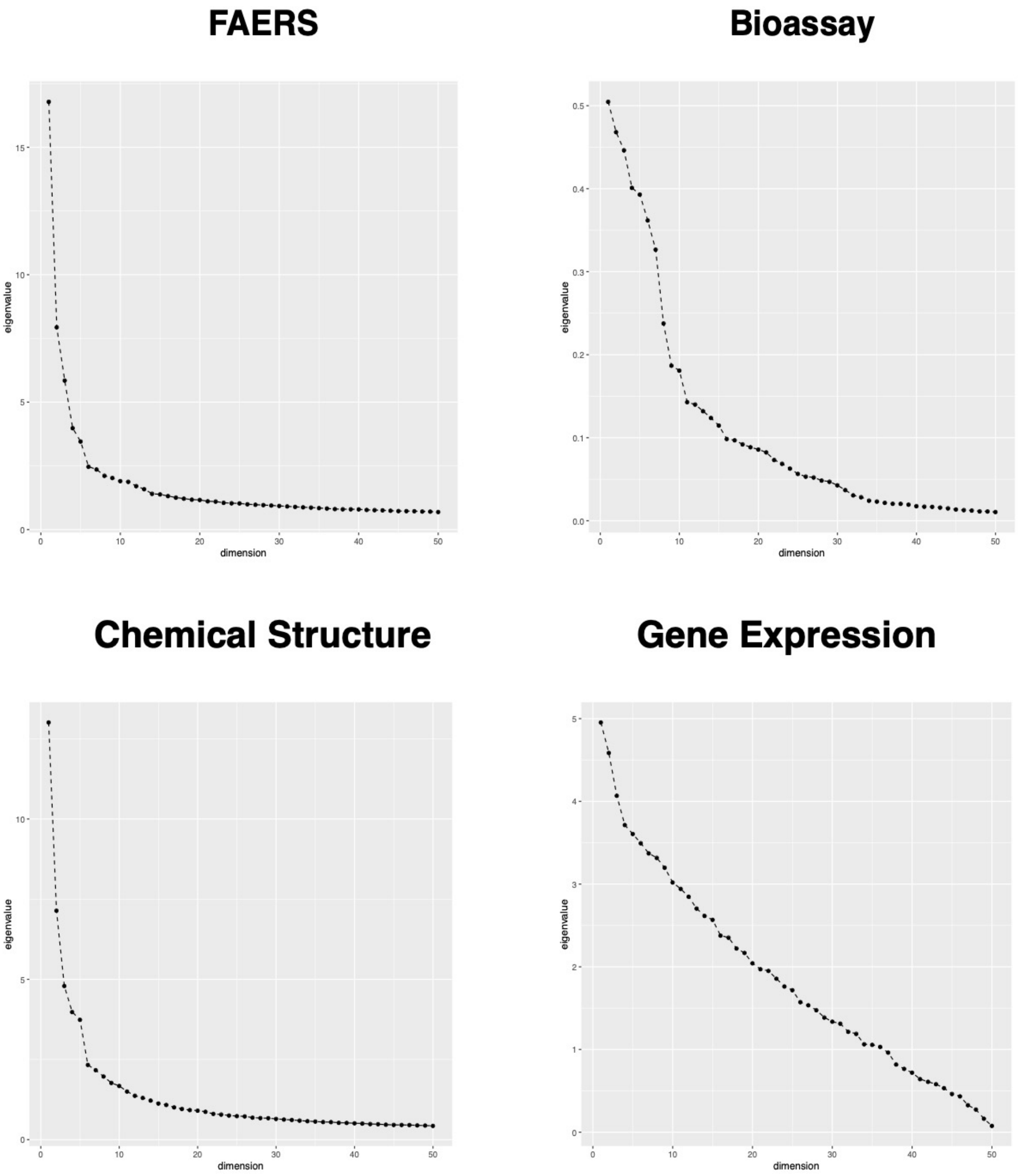

References

ALEXANDROV, L. B., NIK-ZAINAL, S., WEDGE, D. C., CAMPBELL, P. J. & STRATTON, M. R. 2013. Deciphering signatures of mutational processes operative in human cancer. Cell reports, 3, 246-259.

SUBRAMANIAN, A., NARAYAN, R., CORSELLO, S. M., PECK, D. D., NATOLI, T. E., LU, X., GOULD, J., DAVIS, J. F., TUBELLI, A. A. & ASIEDU, J. K. 2017. A next generation connectivity map: L1000 platform and the first 1,000,000 profiles. Cell, 171, 1437-1452. e17.

LAMB, J., CRAWFORD, E. D., PECK, D., MODELL, J. W., BLAT, I. C., WROBEL, M. J., LERNER, J., BRUNET, J.-P., SUBRAMANIAN, A. & ROSS, K. N. 2006. The Connectivity Map: using gene-expression signatures to connect small molecules, genes, and disease. science, 313, 1929-1935.

AKINCI, E., CHA, M., LIN, L., YEO, G., HAMILTON, M. C., DONAHUE, C. J., BERMUDEZ-CABRERA, H. C., ZANETTI, L. C., CHEN, M. & BARKAL, S. A. 2020. Elucidation of remdesivir cytotoxicity pathways through genome-wide CRISPR-Cas9 screening and transcriptomics. BioRxiv.

WANG, B., MEZLINI, A. M., DEMIR, F., FIUME, M., TU, Z., BRUDNO, M., HAIBE-KAINS, B. & GOLDENBERG, A. 2014. Similarity network fusion for aggregating data types on a genomic scale. Nature methods, 11, 333-337.

HUANG, L., LUO, H., LI, S., WU, F.-X. & WANG, J. 2021. Drug–drug similarity measure and its applications. Briefings in Bioinformatics, 22, bbaa265.

BU, D., LUO, H., HUO, P., WANG, Z., ZHANG, S., HE, Z., WU, Y., ZHAO, L., LIU, J. & GUO, J. 2021. KOBAS-i: intelligent prioritization and exploratory visualization of biological functions for gene enrichment analysis. Nucleic acids research, 49, W317-W325.

AI, C. & KONG, L. 2018. CGPS: a machine learning-based approach integrating multiple gene set analysis tools for better prioritization of biologically relevant pathways. Journal of Genetics and Genomics, 45, 489-504.
